# Efficient workflow scheduling in fog-cloud collaboration using a hybrid IPSO-GWO algorithm

**DOI:** 10.1038/s41598-025-34462-w

**Published:** 2026-01-30

**Authors:** Samar Awad, Marwa Gamal, Khaled Abd El Salam, Rehab F. Abdel-Kader

**Affiliations:** 1 Electrical Engineering Department, Computer and Control Branch, Faculty of Engineering, Suez Canal University, Ismailia, 41522 Egypt; 2 Department of Information System, College of Information Technology, Misr University for Science and Technology (MUST), 6th of October City , 12566 Egypt; 3 Faculty of Engineering Technology, ElSewedy University of Technology, Cairo, 11757 Egypt; 4 Faculty of Engineering, Port Said University, Port Said, 42523 Egypt

**Keywords:** Fog-Cloud computing, Grey wolves optimization (GWO), Offloading, Particle swarm optimization (PSO), Scientific workflow, And workflow scheduling, Engineering, Mathematics and computing

## Abstract

With the rapid advancement of fog-cloud computing, task offloading and workflow scheduling have become pivotal in determining system performance and cost efficiency. To address the inherent complexity of this heterogeneous environment, a novel hybrid optimization strategy is introduced, integrating the Improved Particle Swarm Optimization (IPSO) algorithm, enhanced by a linearly decreasing inertia weight, with the Grey Wolf Optimization (GWO) algorithm. This hybridization is not merely a combination but a synergistic fusion, wherein the inertia weight adapts dynamically throughout the optimization process. Such adaptation ensures a balanced trade-off between exploration and exploitation, thereby mitigating the risk of premature convergence commonly observed in standard PSO. To assess the effectiveness of the proposed IPSO-GWO algorithm, extensive simulations were carried out using the FogWorkflowSim framework—an environment specifically developed to capture the complexities of workflow execution within fog-cloud architectures. Our evaluation encompasses a range of real-world scientific workflows, scaling up to 1000 tasks, and benchmarks the performance against PSO, GWO, IPSO, and the Gravitational Search Algorithm (GSA). The Analysis of Variance (ANOVA) is employed to substantiate the results. The experimental results reveal that the proposed IPSO-GWO approach consistently outperforms existing baseline methods across key performance metrics, including total cost, average energy consumption, and overall workflow execution time (makespan) in most scenarios, with average reductions of up to 26.14% in makespan, 37.73% in energy consumption, and 12.52% in total cost Beyond algorithmic innovation, this study contributes to a deeper understanding of workflow optimization dynamics in distributed fog-cloud systems, paving the way for more intelligent and adaptive task scheduling mechanisms in future computing paradigms.

## Introduction

The rapid development of communication networks has significantly driven the expansion of the IoT^1^. This expansion led to an exponential increase in real-time data generation, and processing demands across various domains, including smart cities, healthcare, industrial automation, and environmental monitoring. It demands increasingly efficient computational support from both fog and cloud infrastructures^2^. Although Cloud Computing (CC) offers access to robust resources, it often falls short in addressing the real-time requirements of specific IoT applications^3^. Rather than replacing the cloud, fog computing works alongside it to manage various task durations and computational needs. Cisco introduced Fog Computing (FC)^4^, which emerged as a distributed transformative computing model aimed at bridging the gap between cloud infrastructure and the IoT. By bringing storage and computational resources closer to the network edge, this paradigm effectively tackles challenges such as network congestion, excessive communication costs, and delays in data transmission^5^. Its capability of processing the data locally not only enhances efficiency but also empowers IoT systems to deliver faster, more responsive, and more reliable services.

Workflows representing real-world applications require efficient offloading and careful scheduling to ensure timely and cost-effective execution. Scientific workflows are intricate networks of interdependent tasks and calculations, purposefully designed to achieve specific scientific endeavors. These real-time workflows are often visualized as Directed Acyclic Graphs (DAGs), where nodes signify the tasks and the edges illustrate the relationships between them^6^. Early implementations of these workflows were predominantly hosted on distributed systems and High Performance Computing (HPC) platforms^7,8^, focusing primarily on resource distribution, workload balancing, and execution management. In recent years, the evolution of CC has revolutionized scientific workflows^9^, offering centralized resources through a client-server model and operating on a pay-as-you-go basis^10^. This transition has been driven by CC’s affordability, speed, scalability, and advanced capabilities like virtualization and batch processing. Such attributes have made CC an essential tool for researchers tackling computation- and communication-heavy workflows. High computational and communication costs often hinder task allocation in cloud environments. FC addresses these issues by enabling real-time, localized processing, complementing CC. The integrated IoT-Fog-Cloud (IFC) environment^11^ enhances workflow efficiency, but dynamic demands and resource constraints make task scheduling a complex challenge. The task scheduling challenge in the IFC environment is classified as an NP-hard problem, which entails assigning available Virtual Machines (VMs) to individual workflow tasks within the system architecture, and can be addressed through multi-objective optimization methods^12^.

Many studies have been presented to solve the workflow scheduling problem using optimization techniques that are based on population, such as PSO and Genetic Algorithm (GA)^13–15^. The main performance metrics that have been the focus of many studies are total cost, average energy consumption, and the total time required to complete the workflow (makespan). According to a recent in-depth study on various workflow scheduling methods aimed at fulfilling Quality of Service requirements^16^, PSO-based algorithms are among the most commonly utilized in this domain. Another study also explored an advanced variant of PSO, referred to as multi-swarm particle swarm optimization, for workflow scheduling^17^. As outlined by the No-Free-Lunch (NFL) theorem^18^, in essence, no optimization algorithm can outperform all others across every problem domain; its effectiveness varies depending on the specific problem set. Each swarm intelligence method faces varying challenges, including premature speed convergence, reduced optimization performance, slow convergence speed, and a tendency to quickly reach local optima^19^. The algorithm’s ability to achieve a satisfactory balance between exploration and exploitation at each stage of the search process is one of the factors contributing to these challenges^20^. The optimization based on population for workflow scheduling in the IFC environment is categorized into two primary groups. The first type^21,22^ relies on Pareto optimality techniques to derive a set of optimal solutions. These techniques consider that task scheduling may involve conflicting optimization objectives. Regardless of the shape of the Pareto front (whether convex or not), a set of non-dominated optimal solutions achieves a balance between these conflicting objectives. However, in practical applications, it is necessary to use a posteriori techniques to select the best compromise solution from the set of Pareto optimal solutions^23^. The second group integrates multiple numbers of objectives into a single function of the sum of weighted objectives, as in^17,24,25^. It can be optimized using single-objective techniques such as GWO, PSO, and similar methods. These techniques struggle to explore it effectively if the Pareto front is non-convex in minimization problems; otherwise, this limitation does not exist^26^. Since the Pareto fronts for solving the workflow scheduling problem are mostly classified as convex, the weighted sum method is used in this work. In the workflow scheduling problem, this method generates optimal solutions effectively without the need for a subsequent optimization step to select the best compromise schedule.

The motivation for this work arises from the critical need to balance exploration and exploitation in metaheuristic optimization for workflow scheduling. Exploration enhances solution diversity and avoids local optima, while exploitation refines promising areas of the search space for faster convergence. Excessive exploration slows convergence, while excessive exploitation risks premature stagnation. To address this trade-off, we propose a hybrid Improved Particle Swarm Optimization–Grey Wolf Optimization (IPSO-GWO) algorithm.

In summary, the main idea of this paper is to design and validate a hybrid IPSO-GWO algorithm for workflow scheduling in heterogeneous fog–cloud environments. The motivation is to overcome the limitations of single-algorithm approaches that either converge prematurely or lack sufficient diversity. The objectives are threefold: (1) to minimize makespan, energy consumption, and execution cost in large-scale scientific workflows; (2) to achieve a more effective balance between exploration and exploitation; and (3) to provide a scalable and adaptive scheduling solution for IFC systems. The novelty of the proposed IPSO-GWO lies in its synergistic integration of IPSO and GWO: IPSO enhances exploitation by dynamically adapting inertia weight for refined local search, while GWO strengthens exploration to maintain diversity and avoid local optima. Together, these mechanisms enable faster convergence and superior performance compared to traditional algorithms.

The key contributions of this study are presented as follows:

1. This study focuses on the heterogeneous IFC system and highlights the challenges associated with resource management. It is a hierarchical architecture, and it has been modeled to serve workflow scheduling for real-time applications with computationally intensive or time-sensitive characteristics. It integrates IoT devices, FC, and CC to optimize data processing, storage, and communication.
2. In this study, a weighted sum-based objective functions were considered, which includes the total completion time for workflow tasks, total cost, and the average energy consumption. It was found that energy consumption is rarely considered as an objective in many research studies^21,27^.
3. The study proposes a novel hybrid task scheduling algorithm (IPSO-GWO). It combines the strengths of both techniques to achieve better convergence rates and more accurate solutions. It can achieve an optimal balance between searching broadly for solutions (exploration) and refining existing solutions (exploitation).
4. In order to simulate the heterogeneous IFC system, this study incorporates five real-time scientific workflows: SIPHT, LIGO, CyberShake, Epigenomics, and Montage, with the number of tasks reaching up to 1000.
5. To measure the behavior of the proposed IPSO-GWO algorithm for workflow scheduling problems, we conducted extensive simulations and compared it with several population-based meta-heuristic algorithms, including IPSO, PSO, GWO, and GSA. The goal of this analysis was to showcase the capability of the proposed algorithm in solving the workflow scheduling problem efficiently.

This study is implemented using FogWorkflowSim^27^. The remainder of the paper is structured as follows: Section “Related work” provides a literature review on task scheduling algorithms and offloading strategies. Section “Background” outlines workflow scheduling and the associated performance metrics. Section “Proposed method” details the concept of workflows and introduces the proposed method. Section “Evaluation of performance” presents and analyzes the experimental results, while Section “Conclusion and future work” concludes the research.

## Related work

Due to the increasing complexity of tasks in heterogeneous fog–cloud computing environments (IFC), along with the urgent need for efficient resource management, workflow scheduling emerges as a vital research focus that attracts significant attention from researchers because of its direct impact on system performance and service sustainability. Several algorithms have been introduced to tackle the challenges associated with workflow scheduling, aiming to enhance execution time, reduce energy consumption, and lower overall costs. The upcoming sections provide an in-depth overview of the most influential strategies and key findings presented in recent research efforts within this evolving domain.

In^28^ three novel task offloading strategies tailored for IoT–fog–cloud environments are introduced, emphasizing the requirements of real-time applications. The first strategy, Latency Centric Offloading (LCO), is designed to efficiently manage tasks that demand minimal response time. The second, Energy-Based Offloading (EBO), focuses on energy conservation while boosting performance for resource-heavy computational tasks. The third, Efficient Offloading, seeks to achieve an optimal equilibrium between reducing latency and conserving energy, thus enhancing the effective use of available resources. In^29^, a comparison between cloud and fog-cloud collaboration by utilizing the well-known PSO for workflow scheduling is conducted. A Multi-Swarm PSO algorithm was introduced for task scheduling in a fog-cloud environment^17^, focusing on optimizing four key objectives: energy consumption, cost, makespan, and load balancing. This approach addresses issues such as premature convergence and demonstrates improved performance over traditional methods like standard PSO and GA. An Enhanced Particle Swarm Optimization (EPSO) is proposed in^30^. It demonstrates superior performance in minimizing makespan, energy consumption, and cost compared to traditional methods like PSO, Max-Min, and Round-Robin. EPSO optimizes the allocation of tasks to fog servers, thereby reducing latency and improving Quality of Service (QoS).

In^31^, a multi-objective Improved Particle Swarm Optimization (IPSO) algorithm is proposed. IPSO outperforms standard PSO in workflow task allocation efficiency. The study in^32^ investigates meta-heuristic task scheduling by employing an Ant Colony Optimization (ACO) algorithm in fog-cloud environments. The main goal is to reduce response time, enhance resource utilization, and improve task mobility across fog nodes. The proposed ACO-based approach demonstrates superior performance compared to conventional scheduling algorithms such as RR, Shortest Job First (SJF), and FCFS. An Improved Zebra Algorithm (ImZP) was introduced for task scheduling based on priority awareness in fog-cloud environments in^33^. This approach enhances exploration capabilities by integrating differential evolution, enabling an optimal task scheduling algorithm based on multi-objective functions such as cost, task priority, and execution time. The study in^34^ presented the Improved Butterfly Optimization Algorithm (IBOA) for scheduling tasks in hybrid cloud-fog environments. By dynamically updating computation and communication costs, the IBOA enhanced adaptability and achieved substantial reductions in total, communication, and computation costs when compared to legacy scheduling techniques. A GWO-based algorithm was introduced for task scheduling in cloud-fog environments in^35^. This approach enhanced workflow performance by avoiding local optima and improving QoS parameters, while also reducing energy consumption and costs in hybrid cloud-fog architectures.

A method was introduced to schedule tasks in cloud-fog computing^36^, employing a Binary Particle Swarm Optimizer (BPSO) that incorporated a modified sigmoid function along with a logarithmically decreasing inertia weight strategy. This approach aimed to enhance the makespan and minimize load imbalance. The Multi-objective Artificial Algae (MAA) was designed for scheduling workflows in heterogeneous Fog-Cloud environments^37^. It optimized execution time, energy consumption, and total cost while addressing task interdependencies by preprocessing and prioritizing bottleneck tasks. A “Differential Evolution-Grey Wolf Optimization” (DE-GWO) was developed^38^ to enhance the scheduling of workflows in cloud-fog computing systems. This approach combined Differential Evolution (DE) with GWO to address the low accuracy and slow convergence issues found in traditional GWO algorithms. By combining DE, the algorithm enhances the evolutionary process of the ‘wolves,’ leading to faster convergence and better accuracy. Additionally, the study introduces an objective function that balances three key factors: energy consumption, cost, and makespan. A study in^39^ introduced a hybrid “Particle Whale Optimization Algorithm” (PWOA) to improve the scheduling of workflow in cloud-fog computing. This algorithm combines features from PSO and the WOA to enhance both exploration and exploitation capabilities. The primary goal is to reduce Total Execution Cost (TEC) and Total Execution Time (TET) by addressing task allocation challenges through improved optimization techniques. In study^40^, Bezdan et al. develop a novel approach in the form of a hybrid algorithm inspired by bat behavior. This algorithm effectively leverages swarm intelligence principles to tackle the challenges of multi-objective task scheduling, with a primary focus on reducing the time required to search for optimal solutions.

In^41^, the study introduced a hybrid algorithm that combined GA and modified PSO to enhance multiple-objective task scheduling in fog computing. By merging the strengths of GA and PSO, the hybrid approach effectively explores and exploits the specified search space, resulting in better performance than traditional methods that rely on a single algorithm. A Shark Search Krill Herd Optimization (SSKHOA) method for task scheduling in fog-cloud computing was proposed in^42^. The SSKHOA algorithm integrates both the shark search and the krill herd algorithms to improve both global and local search capabilities during optimization. By emulating the swarm intelligence of krill herds and the predator-prey behavior of sharks, SSKHOA efficiently explores the solution space and identifies near-optimal task schedules. This approach enhances performance in cloud-fog computing environments. A “Hybrid Flamingo Search with Genetic Algorithm” (HFSGA) to improve task scheduling^43^. This study is designed to measure its effectiveness; the performance of HFSGA is tested using seven basic benchmark optimization functions and compared with well-established other algorithms. This comparison demonstrates the advantages of HFSGA in achieving better task scheduling outcomes. An Ant Grey Wolf Optimization (AGWO) for task scheduling in cloud-fog computing was introduced in this study^44^. AGWO integrates the strengths of ACO and GWO to improve the speed and quality of finding optimal solutions. AGWO achieves efficient task scheduling in the fog-cloud environment. In^45^, it introduced a novel hybrid algorithm called HPSOGWO, which combined PSO and GWO. HPSOGWO’s main goal is to optimize total execution cost and total execution time. Experimental results demonstrate that the introduced algorithm outperforms other methods like PSO, “Heterogeneous Earliest Time First” (HEFT), ACO, and RR, significantly minimizing execution cost and time.

The TM-MOAOA approach^46^ for task scheduling in fog-cloud environments integrates the Technique for Order of Preference by Similarity to Ideal Solution (TOPSIS) with multi-objective Archimedes optimization. This two-stage method aims to enhance task allocation efficiency while addressing critical factors such as energy consumption, latency, and resource utilization. Similarly, a hybrid scheduling algorithm based on the Markov chain was proposed to achieve load balancing in fog–cloud environments, resulting in improved response time^47^. This approach leverages the dynamic nature of fog computing to optimize resource allocation and minimize latency, particularly for time-sensitive applications. ALBLA employs learning automata to achieve adaptive load balancing within edge–cloud networks^48^, demonstrating the effectiveness of lightweight learning methods in decentralized environments. This study in^49^ analyzes the Google Cluster Dataset to evaluate task execution characteristics in large-scale cloud environments. It focuses on understanding the impact of memory, processor performance, task constraints, and priority on task execution time and rescheduling behavior. A hybrid Particle Swarm Optimization–Simulated Annealing^49^ (PSO–SA) approach is proposed to optimize task scheduling for IoT workflows in fog–cloud environments. The proposed method efficiently prioritizes interdependent tasks, reducing both energy consumption and makespan compared to the IKH-EFT baseline. Simulation results demonstrate that PSO–SA achieves an average improvement of 5% in energy efficiency and 9% in makespan reduction, highlighting its effectiveness in complex IoT workflow scenarios. A multi-objective workflow scheduling approach^50^ in fog–cloud environments using the Krill Herd Optimization (KHO) algorithm to balance energy consumption, makespan, and monetary cost. By leveraging the EFT (Earliest Finish Time) technique and dynamic resource voltage and frequency, the method achieves efficient task allocation and reduced energy use. Simulation results demonstrate that the proposed approach improves the makespan by 9.9%, 8.7%, and 6.7% compared to IHEFT, HEFT, and IWO-CA, respectively, while also minimizing energy consumption and cost. Despite extensive research on cloud–fog workflow scheduling, several challenges in scheduling algorithms remain unresolved, and the key contributions of existing studies are summarized in Table 1.

**Table 1:** Task scheduling algorithms literature Review.

References	Performance metrics	Improvements	Limitations
^28^	Makespan, Energy consumption, and total cost	Developing tailored offloading methods to meet the needs of real-time applications	It does not address the scalability of the proposed methods in larger or more complex IoT environments
^29^	Makespan, Energy consumption, and total cost	It presents several insights into improving workflow scheduling algorithms	The need for a broader evaluation of optimization algorithms in workflow scheduling within cloud-fog environments
^30^	Makespan, Energy consumption, and total cost	It focuses on enhanced performance, minimized makespan, energy efficiency, stability, and adaptability to various workflows	Only the makespan is considered as the objective function, which primarily caters to delay-sensitive applications
^31^	Makespan, Energy consumption, and total cost	An improvement in makespan, energy consumption, and total cost compared to PSO with task varying	It may still be susceptible to getting trapped in local optima, especially in complex search spaces, and has only been compared against PSO
^32^	Response time	Reduce overall response time	Traditional scheduling algorithms like FCFS, SJF, and RR are used for comparing the performance
^33^	priority, availability, makespan, energy consumption, cost, and success rate	It demonstrates its efficiency in optimizing task scheduling and attains significant results for these metrics	The limited real-world testing may constrain the broader applicability and generalizability of the results
^34^	Total cost	It shows substantial reductions in multiple task execution costs—19.65% in total cost, 18.28% in communication cost, and 25.41% in computation cost.	Only the cost is taken into account as the objective criterion
^35^	Makespan, Energy consumption, and total cost	It reduces energy consumption and cost	It uses dynamic weights to improve performance, but tuning them adds complexity
^36^	Makespan	Reduce the makespan	Other QoS metrics aren’t taken into consideration as cost and energy consumption
^37^	Execution times, energy consumption, and overall costs	The average result shows a reduction in execution time, energy consumption, and total cost of about 43%, 28%, and 10%, respectively	Real-time implementation and analysis of the proposed algorithm have not been addressed due to cost constraints
^38^	Makespan, Energy consumption, and total cost	Achieving a balance between exploration and exploitation and improving scheduling efficiency	It does not consider other important objectives such as reliability, fault tolerance, and deadlines
^39^	Total Execution Time and Total Execution Cost	It outperforms PSO and WOA in minimizing both Total Execution Time and Total Execution Cost	Energy consumption is not considered, and it has only been compared with PSO and WOA
^40^	Makespan and Cost	By incorporating a QRBL mechanism and combining BA with ABC, the Hypervolume metric is significantly improved	Not taking into consideration several objectives as Energy consumption
^41^	Makespan, Energy consumption, and total cost	Improve QoS metrics by reducing makespan, energy consumption, and total cost	Its scalability is limited and needs to be extended to encompass multiple data centers across diverse settings
^42^	Makespan and Execution Time	Increase task success rate by 34%, reduce execution time by 36%, and lower makespan time by 54% compared to baseline methods	Energy consumption and cost are not considered, and further studies must validate these results across diverse scenarios and datasets
^43^	Makespan, cost, and Percentage of deadline-satisfied tasks	It yields better results for the task scheduling process.	Energy consumption is not considered
^44^	Makespan and Cost	AGWO outperforms competing methods in task scheduling, cutting makespan by 42% and reducing costs by 36%	Parameter Sensitivity, Scalability, Convergence to Suboptimal Solutions, and Limited Problem-Specific Adaptability
^45^	Makespan and Cost	It lowers execution time and cost relative to other baseline algorithms	It overlooks energy consumption and relies solely on cloud computing instead of a three-tier network
^46^	Energy consumption, latency, and resource utilization.	More efficient resource utilization	The two-stage nature (TOPSIS + MOAOA) may introduce extra overhead in decision-making / optimization.
^47^	Makespan, Delay, Performance Improvement Rate (PIR), and Throughput	Effectively minimizes makespan and delay, while maximizing throughput and PIR, making it well-suited for real-time IoT applications in fog-cloud environments.	Challenges in Large-Scale Deployments and Increased Processing Overhead
^48^	Average service time, Task waiting time, Network traffic, Number of successful task executions on edge servers	Reduced Latency, Enhanced Resource Management, and the hybrid architecture supports scalability	Diverse technologies and standards can complicate integration, leading to potential inefficiencies
^49^	Task execution time, Rescheduling frequency, Task priority, Number of task constraints	It provides a deeper understanding of how task constraints and priorities correlate with rescheduling behavior, which can inform better scheduling policies	While memory’s influence is strongly highlighted, the interplay with other resource types or network effects may not be deeply explored
^50^	Energy consumption, Makespan, Task priority satisfaction, Deadline adherence	The proposed hybrid PSO–SA algorithm is shown to reduce energy consumption by ~ 5% on average compared to the IKH-EFT baseline. It also reduces the makespan by ~ 9% on average.	The method might struggle with deadline violation under high load, since optimizing energy and makespan jointly with deadlines is challenging
^51^	Makespan, Energy consumption, Monetary cost, Customer satisfaction, Response time	It effectively reduces energy consumption and monetary cost, enhancing the overall efficiency of fog–cloud resource utilization	The study lacks a comparative scalability analysis on very large datasets or heterogeneous fog nodes with differing capabilities

## Background

This section begins with a systematic presentation of the problem formulation, followed by a detailed analysis of three objective functions adopted within the framework of the proposed algorithm. It also includes an explanation of the theoretical foundations and core techniques derived from the Improved Particle Swarm Optimization (IPSO) and Grey Wolf Optimizer (GWO) algorithms, which contributed to shaping the practical structure of the developed algorithm.

### System description

**Fig. 1:**
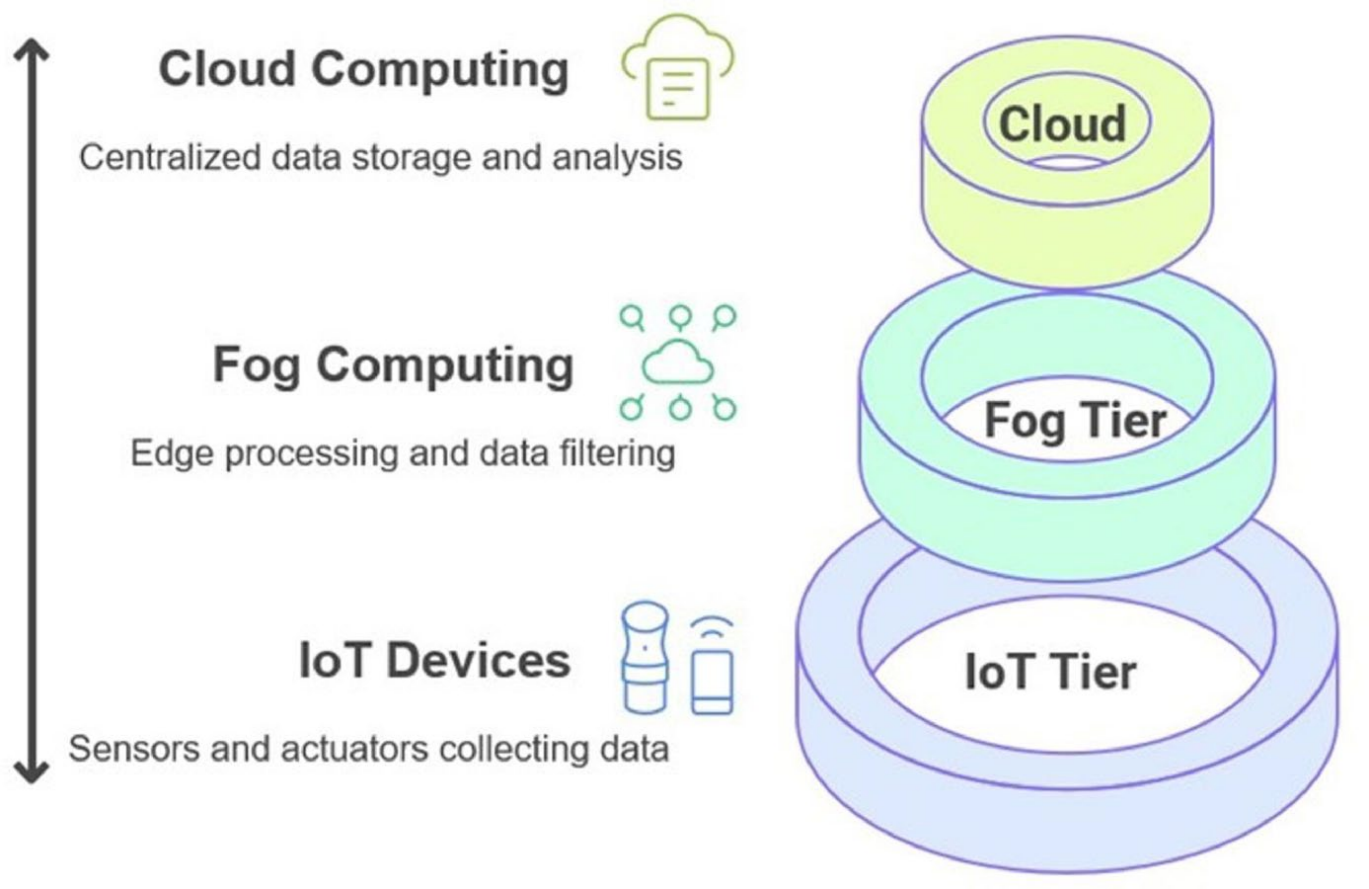
IFC System Hierarchy.

The proposed IFC system is organized into a three-layer architecture as shown in Fig. 1, consisting of the IoT layer, the Fog layer, and the Cloud layer, each playing a distinct role in workflow execution.

- IoT Layer: This layer is closest to end-users and is responsible for processing latency-sensitive tasks. Due to limited computational capacity, only lightweight or real-time components of workflows are executed at this level, thereby reducing response delay and minimizing the communication overhead to higher layers.
- Fog Layer: Situated between the edge and the cloud, fog devices provide intermediate computational and storage resources. They serve as aggregators that offload part of the computational burden from the cloud while offering lower latency compared to remote data centers. Workflow tasks with moderate resource demands or those requiring faster turnaround are scheduled here.
- Cloud Layer: This layer offers virtually unlimited computational power and storage. Tasks with high computational intensity or large-scale data requirements are distributed to cloud resources. However, executing tasks in the cloud often incurs higher communication latency and financial cost, making it less favorable for delay-sensitive applications.

In this architecture, workflow tasks are distributed according to their resource demands and QoS requirements. Tasks are mapped while respecting DAG dependencies, such that:

1. Latency-sensitive tasks are executed at the edge when possible.
2. Intermediate-demand tasks are offloaded into fog devices to balance execution time and energy consumption.
3. Computationally intensive tasks are delegated to the cloud to leverage its high processing capacity, despite the higher cost.

This hierarchical distribution enables a balanced utilization of resources across the three layers, thereby reducing makespan, cost, and energy consumption while ensuring that task dependencies remain preserved throughout execution. Moreover, the task distribution strategy is strongly influenced by the proposed hybrid IPSO-GWO scheduling algorithm and the design of the fitness function, which integrates the selected performance criteria. As will be detailed in the following sections, these two components play a central role in determining how tasks are mapped to resources, ultimately shaping the overall efficiency of workflow execution.

### Problem formulation

**Fig. 2:**
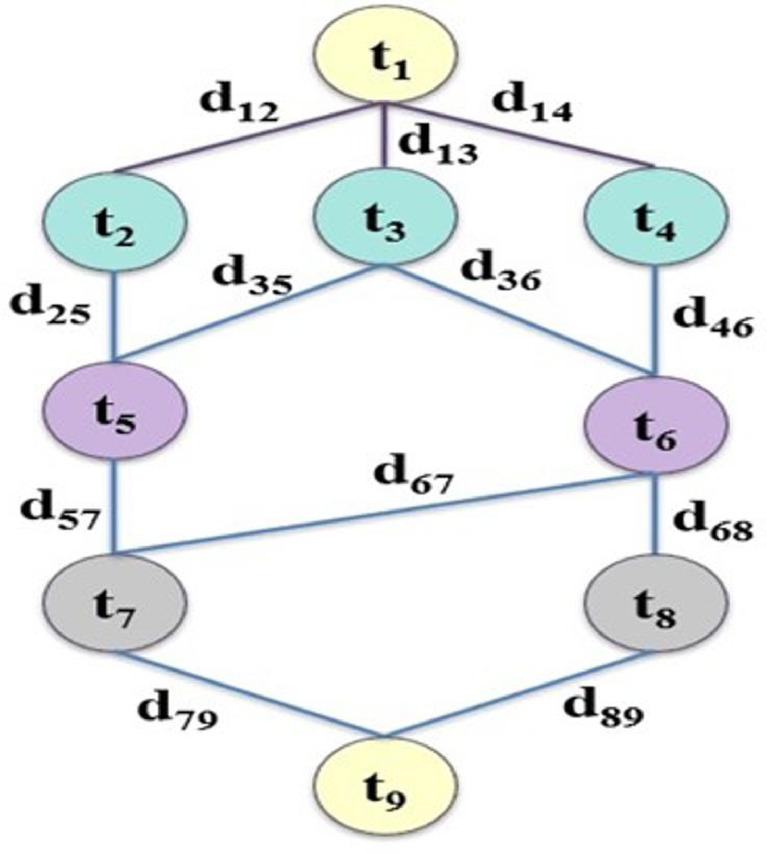
An example of nine tasks in a DAG-based workflow^30^.

DAGs are often used for modeling scientific workflows and to represent task dependencies^52^. In these graphs, tasks are shown as “nodes”, and the task dependencies are declared as “edges”. This helps visualize the order and relationships of tasks in the workflow. Workflows are crucial for handling the growing computational needs of today’s research. They simplify complex problems by dividing them into smaller, easier-to-manage tasks. This allows researchers to use large amounts of data and available computing resources in cloud environments efficiently. DAG is represented by G = (T, E), where T stands for the vertices (tasks from t_1_ to t_n_) and E represents edges (task dependencies). Each edge, written as d_i, j_ = < t_i_, t_j_> ∈ E, shows the data flow between tasks t_i_ and t_j_, with d_i, j_ indicating the data output size from t_i_ to t_j_. Task tj can only begin after task t_i_ finishes. A non-parent task is called a starting task, and its child is called an ending task. Figure 2 shows an example workflow with nine tasks. Tasks on the same level (shown side by side) can be executed simultaneously. For example, tasks t_2_, t_3_, and t_4_ can all run simultaneously. In an IFC setup, offloading and scheduling workflow applications entail distributing tasks across different computing resources. These resources possess distinct attributes, and the objective is to optimize workflow execution by reducing three key metrics: makespan, energy usage, and overall expenses.

The overall task completion time refers to the time interval (makespan) that begins with the start of the first task and ends with the completion of the last one. This metric serves as a fundamental basis for evaluating the effectiveness of any task scheduling algorithm. In addition, computing costs—along with the expenses of exchanging data between tasks and processing centers—emerge as critical factors influencing system performance. There is often a trade-off between reducing completion time and resource consumption; minimizing execution time typically requires allocating more computing power, which leads to reliance on high-cost, high-performance Cloud Devices (CDs). Moreover, data transmission between different system components, such as Edge Devices (EDs) and central processing units, imposes additional financial burdens on the end user. The study in^16^ highlights a set of Quality of Service (QoS) criteria that should be considered when designing workflow scheduling algorithms. Both execution time and cost contribute indirectly to meeting several of these QoS requirements. Energy consumption, on the other hand, stands out as one of the key challenges for modern computing infrastructures, due to its dual impact on operational costs and environmental footprint. This challenge becomes even more significant with the integration of Fog Devices (FDs) into the system architecture, where managing energy usage becomes a crucial factor for ensuring operational efficiency and sustainability.

To enforce these task dependencies, FogWorkflowSim, which extends WorkflowSim and iFogSim, through the workflow engine ensures that a child task is not scheduled until all its parent tasks have finished execution and data transfer is complete. This includes accounting for parent-to-child communication delays and network costs, while maintaining the topological order of the DAG throughout scheduling^27^. Such dependency-aware execution guarantees correctness while enabling optimization of workflow performance across heterogeneous fog-cloud environments.

### Performance metrics

In this section, multiple objectives are discussed as a weighted sum of objectives function. The main objective of scientific workflow offloading strategies and scheduling algorithms is to effectively manage computational tasks to reduce critical QoS performance metrics while achieving predefined goals. This requires structuring the problem to account for key metrics like makespan, energy consumption, and total cost, which reflect various dimensions of workflow execution efficiency.

1. **Makespan**.

The workflow’s makespan represents the total time required to complete the entire workflow. It is computed as:

1 $$MS = \max FT_{{t_{i} }} ,t_{i} \epsilon T - \min ST_{{t_{i} }} ,t_{i} \epsilon T$$

Where $\mathrm{S}{\mathrm{T}}_{{\mathrm{t}}_{\mathrm{i}}}$ represents the starting time and $\mathrm{F}{\mathrm{T}}_{{\mathrm{t}}_{\mathrm{i}}}$ signifies the task ${\mathrm{t}}_{\mathrm{i}}$ finishing time within a specific workflow.

(2) **Energy consumption**.

Energy consumption^24^ is determined by using both idle and active components, denoted as ${\mathrm{E}}_{\mathrm{i}\mathrm{d}\mathrm{l}\mathrm{e}}$ and ${\mathrm{E}}_{\mathrm{a}\mathrm{c}\mathrm{t}\mathrm{i}\mathrm{v}\mathrm{e}}$ ​, respectively. ​${\mathrm{E}}_{\mathrm{i}\mathrm{d}\mathrm{l}\mathrm{e}}$ refers to the energy used when devices are not processing tasks actively, while ${\mathrm{E}}_{\mathrm{a}\mathrm{c}\mathrm{t}\mathrm{i}\mathrm{v}\mathrm{e}}$ declares the consumed energy during task execution. The energy consumed during the idle period is calculated by using the following formula:

2 $${\mathrm{E}}_{\mathrm{i}\mathrm{d}\mathrm{l}\mathrm{e}}\mathrm{=}\sum\limits_{\mathrm{j}=1}^{\mathrm{m}}\sum\limits_{{\mathrm{i}\mathrm{d}\mathrm{l}\mathrm{e}}_{\mathrm{i},\mathrm{k}}{\epsilon}{\mathrm{I}\mathrm{D}\mathrm{L}\mathrm{E}}_{\mathrm{i},\mathrm{k}}}{\upalpha\:}{\mathrm{f}}_{\mathrm{m}\mathrm{i}\mathrm{n}\:\mathrm{j}}\:{{\mathrm{V}}^{2}}_{\mathrm{m}\mathrm{i}\mathrm{n}\:\mathrm{j}}\:{\mathrm{L}}_{\mathrm{j},\mathrm{k}},$$

Where ${\mathrm{I}\mathrm{D}\mathrm{L}\mathrm{E}}_{\mathrm{i},\mathrm{k}}$ represents a set of idle slots. It consists of multiple idle slots $\mathrm{K}$ on resource $\mathrm{j}$. The minimum operating frequency of the resource $\mathrm{j}$ is represented by ${\mathrm{f}}_{\mathrm{m}\mathrm{i}\mathrm{n}\:\mathrm{j}}$, while ${\mathrm{V}}_{\mathrm{m}\mathrm{i}\mathrm{n}\:\mathrm{j}}$denotes the minimum voltage for the same resource. The duration of the idle period ${\mathrm{i}\mathrm{d}\mathrm{l}\mathrm{e}}_{\mathrm{i},\mathrm{k}}$ is given by ${\mathrm{L}}_{\mathrm{j},\mathrm{k}}$. Thus, active energy consumption is determined using the following formula:

3 $${\mathrm{E}}_{\mathrm{a}\mathrm{c}\mathrm{t}\mathrm{i}\mathrm{v}\mathrm{e}}=\sum\limits_{\mathrm{i}=1}^{\mathrm{n}}{\upalpha\:}{\mathrm{f}}_{\mathrm{i}}\:{{\mathrm{V}}^{2}}_{\mathrm{i}}\:\left(\mathrm{F}{\mathrm{T}}_{\mathrm{t}\mathrm{i}}-\mathrm{S}{\mathrm{T}}_{\mathrm{t}\mathrm{i}}\right),$$

Where the parameter ${\upalpha\:}$ is a fixed constant, while ${\mathrm{f}}_{\mathrm{i}}$ and ${\mathrm{V}}_{\mathrm{i}}$ represent the frequency and supply voltage of the device/resource executing the task $\mathrm{i}$. When a resource is idle, it enters sleep mode, operating at the lowest supply voltage and corresponding reduced frequency. Total Energy ($\mathrm{T}\mathrm{E}$) across the entire IFC system during workflow execution is given by:

4 $$\mathrm{T}\mathrm{E}={\mathrm{E}}_{\mathrm{a}\mathrm{c}\mathrm{t}\mathrm{i}\mathrm{v}\mathrm{e}}+{\mathrm{E}}_{\mathrm{i}\mathrm{d}\mathrm{l}\mathrm{e}}.$$

(3) **Cost**.

This includes costs for both communication and computation. Computational costs apply to EDs, fog, and cloud resources. However, there are no communication costs if tasks are processed on the ED. The computational cost^24^ for using a computing resource $\mathrm{r}$ and the unit processing $\mathrm{p}\mathrm{r}$ is calculated as follows:

5 $${\mathrm{C}\mathrm{E}}_{\mathrm{i}}^{\mathrm{r}}=\mathrm{p}\mathrm{r}.\left(\mathrm{F}{\mathrm{T}}_{\mathrm{t}\mathrm{i}}-\mathrm{S}{\mathrm{T}}_{\mathrm{t}\mathrm{i}}\right),$$

The cost of communication, which accounts for the transmission data expense when transferring a task’s result of a size ${\mathrm{d}}_{\mathrm{i},\mathrm{j}}$ from the device executing the task $\mathrm{i}$ to the device/resource assigned to a task $\mathrm{j}$, is calculated as follows:

6 $${\mathrm{C}\mathrm{C}}_{\mathrm{i},\mathrm{j}}={\mathrm{t}\mathrm{r}\mathrm{c}}_{\mathrm{i},\mathrm{j}}\:.{\mathrm{d}}_{\mathrm{i},\mathrm{j}},$$

Here, the individual communication cost is represented by ${\mathrm{t}\mathrm{r}\mathrm{c}}_{\mathrm{i},\mathrm{j}}$ from the specified resource for the task $\mathrm{i}$ to the allocated desired resource for task j. If both tasks are executed on the same resource, ${\mathrm{t}\mathrm{r}\mathrm{c}}_{\mathrm{i},\mathrm{j}}$ is equal to 0. Finally, for a system with M tasks and $\mathrm{V}\mathrm{M}\mathrm{s}$ computational resources, the Total Cost ($\mathrm{T}\mathrm{C}$) can be computed as follows:

7 $$\mathrm{T}\mathrm{C}=\sum\limits_{\mathrm{i}=1}^{\mathrm{M}}\sum\limits_{\mathrm{j}=1}^{\mathrm{M}}{\mathrm{C}\mathrm{C}}_{\mathrm{i},\mathrm{j}}+\sum\limits_{\mathrm{r}=1}^{\mathrm{V}\mathrm{M}\mathrm{s}}\sum\limits_{\mathrm{j}=1}^{\mathrm{M}}{\mathrm{C}\mathrm{E}}_{\mathrm{i}}^{\mathrm{r}}.$$

The weighted sum approach allows for prioritizing different objectives by adjusting the weights assigned to each one. It is computed and encompasses makespan, energy consumption, and cost. The formula is as follows:

8 $$\mathrm{F}\left(\mathrm{p}\right)=\:{\mathrm{w}}_{1}\mathrm{.MS\:+\:}{\mathrm{w}}_{2}.\mathrm{T}\mathrm{E}{+}{\mathrm{w}}_{3}.\mathrm{T}\mathrm{C}$$

Where $\mathrm{p}$ presents the mapping of a workflow’s M tasks to the $\mathrm{V}\mathrm{M}\mathrm{s}$ available computing resources in the cloud, fog, and edge devices. For example, in PSO, $\mathrm{p}$ is known as a particle. The coefficient weights ${\mathrm{w}}_{1}$, ${\mathrm{w}}_{2\:}$, and $\text{}{\mathrm{w}}_{3\:}$ represent the degree of importance assigned to each objective. Make weights applied in the evaluations of performance are equal to ensure that each objective contributes equally, with w₁ = w₂ = w₃ = 0.2 as in^17^. These equivalent values to assign equal importance to makespan, energy, and cost while leaving flexibility for additional QoS parameters in future extensions. This choice aligns with prior works that have adopted equal weight values to ensure a fair trade-off among multiple objectives, thereby avoiding bias in the optimization process. Moreover, adjusting these weights naturally shifts the optimization focus—for example, a higher ${\mathrm{w}}_{1}$​ favors faster execution, while higher ${\:\mathrm{w}}_{2\:}$​ or ${\:\mathrm{w}}_{3\:}$​ prioritize energy efficiency or cost reduction, respectively.

### Improved particle swarm optimization

Despite PSO’s simplicity, it is highly effective for solving a broad range of optimization problems. However, it is prone to premature convergence. Consequently, various enhancements have been proposed, as discussed in^53^. IPSO builds upon the foundation of PSO, an evolutionary algorithm inspired by the flocking behavior of birds. IPSO is an evolutionary algorithm designed to solve optimization problems through collective intelligence. Particles represent a candidate solution, which navigates the search space to locate the optimal solution. Meanwhile, each particle searches for the optimal solution by considering both its own best-known position ($\mathrm{p}\mathrm{b}\mathrm{e}\mathrm{s}\mathrm{t}$) and the best position found by the entire swarm ($\mathrm{g}\mathrm{b}\mathrm{e}\mathrm{s}\mathrm{t}$).

9 $$\begin{aligned} {\mathrm{V}}_{\mathrm{i}}\left(\mathrm{t}+1\right) & ={\mathrm{w}}_{\mathrm{i}}\times\:{\mathrm{V}}_{\mathrm{i}}\left(\mathrm{t}\right)+{\mathrm{C}}_{1}\times\:{\mathrm{r}}_{1}\times\:\left(\mathrm{p}\mathrm{b}\mathrm{e}\mathrm{s}\mathrm{t}-{\mathrm{X}}_{\mathrm{i}}\left(\mathrm{t}\right)\right) \\ & \quad+{\mathrm{C}}_{2}\times\:{\mathrm{r}}_{2}\times\:\left(\mathrm{g}\mathrm{b}\mathrm{e}\mathrm{s}\mathrm{t}-{\mathrm{X}}_{\mathrm{i}}\left(\mathrm{t}\right)\right) \end{aligned}$$

10 $${\mathrm{X}}_{\mathrm{i}}\left(\mathrm{t}+1\right)={\mathrm{X}}_{\mathrm{i}}\left(\mathrm{t}\right)+{\mathrm{V}}_{\mathrm{i}}\left(\mathrm{t}+1\right)$$

Where ${\mathrm{V}}_{\mathrm{i}}$ is the velocity of the particle $\mathrm{i}$ at iteration $\mathrm{t}$, ${\mathrm{X}}_{\mathrm{i}}$ is the current position of the particle i at iteration t, and ${\mathrm{w}}_{\mathrm{i}}$ is a weighting function. ${\mathrm{r}}_{1}\mathrm{a}\mathrm{n}\mathrm{d}\:{\mathrm{r}}_{2\:}$are random numbers within [0,1],$\:\mathrm{g}\mathrm{b}\mathrm{e}\mathrm{s}\mathrm{t}$ is the global best solution for particle $\mathrm{i}$, $\mathrm{p}\mathrm{b}\mathrm{e}\mathrm{s}\mathrm{t}$ represents the best solution for the particle $\mathrm{i}$, ${\mathrm{C}}_{1}\:\mathrm{a}\mathrm{n}\mathrm{d}\:{\mathrm{C}}_{2}$ are coefficient factors. The IPSO begins by placing the particles randomly in a set of problems. The velocities of particles in each iteration are calculated using Eq. (9). After computing the velocities, the position of particles can be calculated using Eq. (10). The process of changing the particles’ position will continue until it meets an end criterion.

The inertia weight ($\mathrm{w}$) in PSO determines the trade-off between exploration (global search) and exploitation (local refinement). A fixed inertia weight may lead to suboptimal convergence, whereas a dynamic inertia weight, such as IPSO with Linearly Decreasing Inertia Weight (LDIW), can improve performance by gradually shifting the focus from exploration to exploitation. The IPSO algorithm modifies the inertia weight dynamically during the iteration process. It starts with a higher value (${\mathrm{w}}_{\mathrm{b}\mathrm{e}\mathrm{g}\mathrm{i}\mathrm{n}}$) to encourage exploration in the early iterations and gradually decrease to a lower value (${\mathrm{w}}_{\mathrm{e}\mathrm{n}\mathrm{d}}$) to enhance exploitation in later iterations. These improvements typically include:

1. Adaptive inertia weight: dynamically adjusted to encourage exploration in early iterations and exploitation in later iterations.
2. Chaotic/randomized learning factors: avoid stagnation by diversifying the search space.
3. Local search refinements: used to refine solutions around promising regions.

As a result, IPSO achieves faster convergence, improved solution quality, and better avoidance of local optima compared to standard PSO.

The formula is as follows:

11 $${\mathrm{w}}^{\mathrm{t}}\:=\:{\mathrm{w}}_{\mathrm{e}\mathrm{n}\mathrm{d}}+\left({\mathrm{w}}_{\mathrm{b}\mathrm{e}\mathrm{g}\mathrm{i}\mathrm{n}}-{\mathrm{w}}_{\mathrm{e}\mathrm{n}\mathrm{d}}\right)\times\:\frac{\mathrm{m}\mathrm{a}\mathrm{x}\mathrm{I}\mathrm{t}\mathrm{e}\mathrm{r}-\mathrm{I}\mathrm{t}\mathrm{e}\mathrm{r}}{\mathrm{m}\mathrm{a}\mathrm{x}\mathrm{I}\mathrm{t}\mathrm{e}\mathrm{r}}$$

**Fig. 3:**
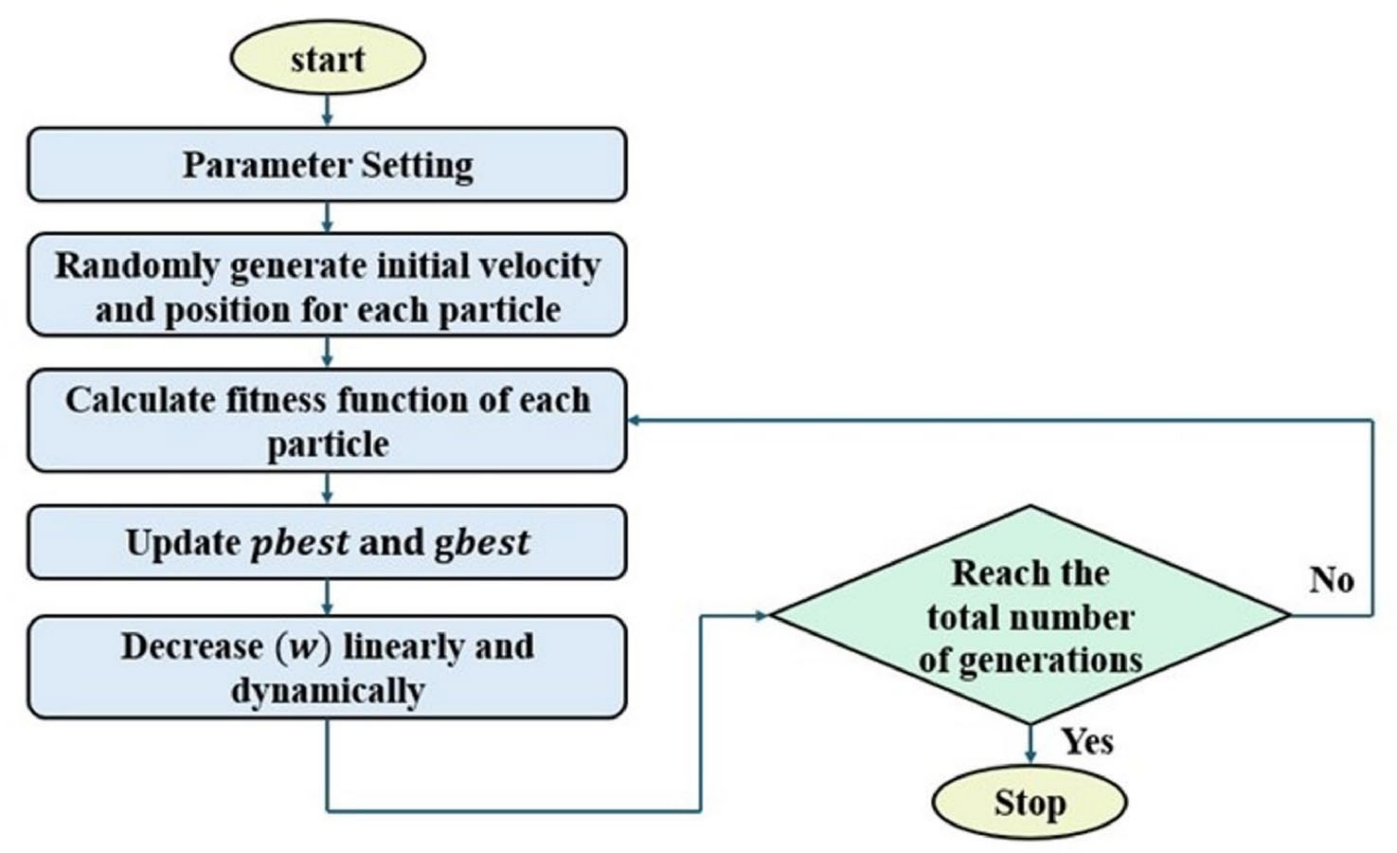
IPSO algorithm flowchart.

Where $\mathrm{m}\mathrm{a}\mathrm{x}\mathrm{I}\mathrm{t}\mathrm{e}\mathrm{r}$ represents the maximum iteration number and $\mathrm{t}$ denotes as the current iteration number. This approach ensures that particles explore diverse solutions in the early phase and refine their search in the later phase, leading to better task scheduling efficiency. The flow chart of IPSO is shown in Fig. 3.

### Grey Wolf optimization

Inspired by the natural behavior of grey wolves, GWO models their pack dynamics and hunting techniques to solve optimization problems, which was introduced in 2014 by Heidari et al.^54^. The GWO algorithm is also used in fog-cloud computing for task scheduling and offloading^25,55^. It is represented hierarchically by four levels of wolves as: alpha $\left({\upalpha\:}\right)$ in the top level, that is the leader for all other wolves, and its position represents the “best solution”. The Beta (${\upbeta\:})$ and delta (${\updelta\:})$ wolves positions denote the second and third-best solutions. The fourth level is named omega (ω) wolves. The wolf pack hierarchy is led by the alpha, who typically makes key decisions but may exhibit democratic behavior by following others; the beta supports the alpha in leadership roles, while the omega occupies the lowest rank. GWO simulates the hunting behavior of wolves in three phases: encircling, hunting, and attacking prey. It models the social hierarchy by assigning roles where α, β, and δ represent the top three solutions guiding the search, while the remaining ω wolves follow their lead to converge on the optimal solution.

#### Encircling prey

For mathematically modeling the encircling process, the following are introduced.

$${\mathrm{X}}_{1}\left(\mathrm{t}\right)={\mathrm{X}}_{{\upalpha\:}}\left(\mathrm{t}\right)-{\mathrm{A}}_{1}\times\:{\mathrm{D}}_{{\upalpha\:}}\left(\mathrm{t}\right),$$

12 $${\mathrm{X}}_{2}\left(\mathrm{t}\right)={\mathrm{X}}_{{\upbeta\:}}\left(\mathrm{t}\right)-{\mathrm{A}}_{2}\times\:{\mathrm{D}}_{{\upbeta\:}}\left(\mathrm{t}\right),$$

$${\mathrm{X}}_{3}\left(\mathrm{t}\right)={\mathrm{X}}_{{\updelta\:}}\left(\mathrm{t}\right)-\mathrm{A}\times\:{\mathrm{D}}_{{\updelta\:}}\left(\mathrm{t}\right),$$

$${\mathrm{D}}_{{\upalpha\:}}=\left|{\mathrm{C}}_{1}\times\:{\mathrm{X}}_{{\upalpha\:}}\left(\mathrm{t}\right)-\mathrm{X}\left(\mathrm{t}\right)\right|,$$

13 $${\mathrm{D}}_{{\upbeta\:}}=\left|{\mathrm{C}}_{2}\times\:{\mathrm{X}}_{{\upbeta\:}}\left(\mathrm{t}\right)-\mathrm{X}\left(\mathrm{t}\right)\right|,$$

$${\mathrm{D}}_{{\updelta\:}}=\left|{\mathrm{C}}_{3}\times\:{\mathrm{X}}_{{\updelta\:}}\left(\mathrm{t}\right)-\mathrm{X}\left(\mathrm{t}\right)\right|,$$

where X$\left(\mathrm{t}\right)$ presents a wolf position. $\mathrm{A}$ and $\mathrm{C}$ (Coefficient vectors) are calculated by Eqs. (14) and (15). ${\mathrm{X}}_{{\upalpha\:}}$, ${\mathrm{X}}_{{\upbeta\:}}$, and ${\mathrm{X}}_{{\updelta\:}}$ describe the best solution of the first three best solutions. ${\mathrm{D}}_{{\upalpha\:}},{\mathrm{D}}_{{\upbeta\:}},$and ${\mathrm{D}}_{{\updelta\:}\:}$describe the distance vectors between the current position and each of the top wolves.

14 $$\mathrm{C}=2\times\:{\mathrm{r}}_{2},$$

15 $$\mathrm{A}=2\times\:\mathrm{a}\times\:{\mathrm{r}}_{1}-\mathrm{a}$$

Here, ${\mathrm{r}}_{1}$, ${\mathrm{r}}_{2}$ are constant numbers in the interval (0, 1), and the vector $\mathrm{a}$ are linearly decreased throughout iterations from 2 to 0 using the following:

16 $$\:\:\:\:\:\:\:\:\mathrm{a}\left(\mathrm{t}\right)=2-\frac{2\times\:\mathrm{t}}{{\mathrm{I}}_{\mathrm{m}\mathrm{a}\mathrm{x}}}$$

#### Hunting

Each grey wolf’s position represents a candidate solution, while the prey signifies the optimal solution. The algorithm assumes that the top three wolves—α, β, and δ—have the best knowledge of the prey’s location and guide the rest (ω wolves), who adjust their positions accordingly. To simulate this behavior, GWO retains the top three solutions and directs the remaining agents to update their positions within a region bounded by these leaders. While α usually leads the hunt, β and δ may also contribute. Although the exact position of the prey is unknown, it is estimated through the coordinated actions of the top wolves, allowing the rest to converge around the optimal area through randomized position updates within the defined circle.

17 $${\mathrm{X}}_{\mathrm{i}-\mathrm{G}\mathrm{W}\mathrm{O}}\left(\mathrm{t}+1\right)=\frac{{\mathrm{X}}_{1}\left(\mathrm{t}\right)+{\mathrm{X}}_{2}\left(\mathrm{t}\right)+{\mathrm{X}}_{3}\left(\mathrm{t}\right)}{3}$$

where ${\mathrm{X}}_{\mathrm{i}-\mathrm{G}\mathrm{W}\mathrm{O}}$represents the position of the grey wolf in the next iteration.

#### Attacking prey (exploitation)

After stopping moving for the victim, the wolves attack it to terminate the hunt. We decrease the value to mathematically emulate approaching the prey. Notice how this reduces the fluctuation range of r. In other words, during the duration of iterations, r is reduced from 2 to 0 and is a random value.

#### Search for prey (exploration)

Grey wolves first utilize the ${\upalpha\:}$, ${\upbeta\:}$, and ${\updelta\:}$ positions for their search guidance. They disperse to search for prey and later regroup to launch an attack.

**Fig. 4:**
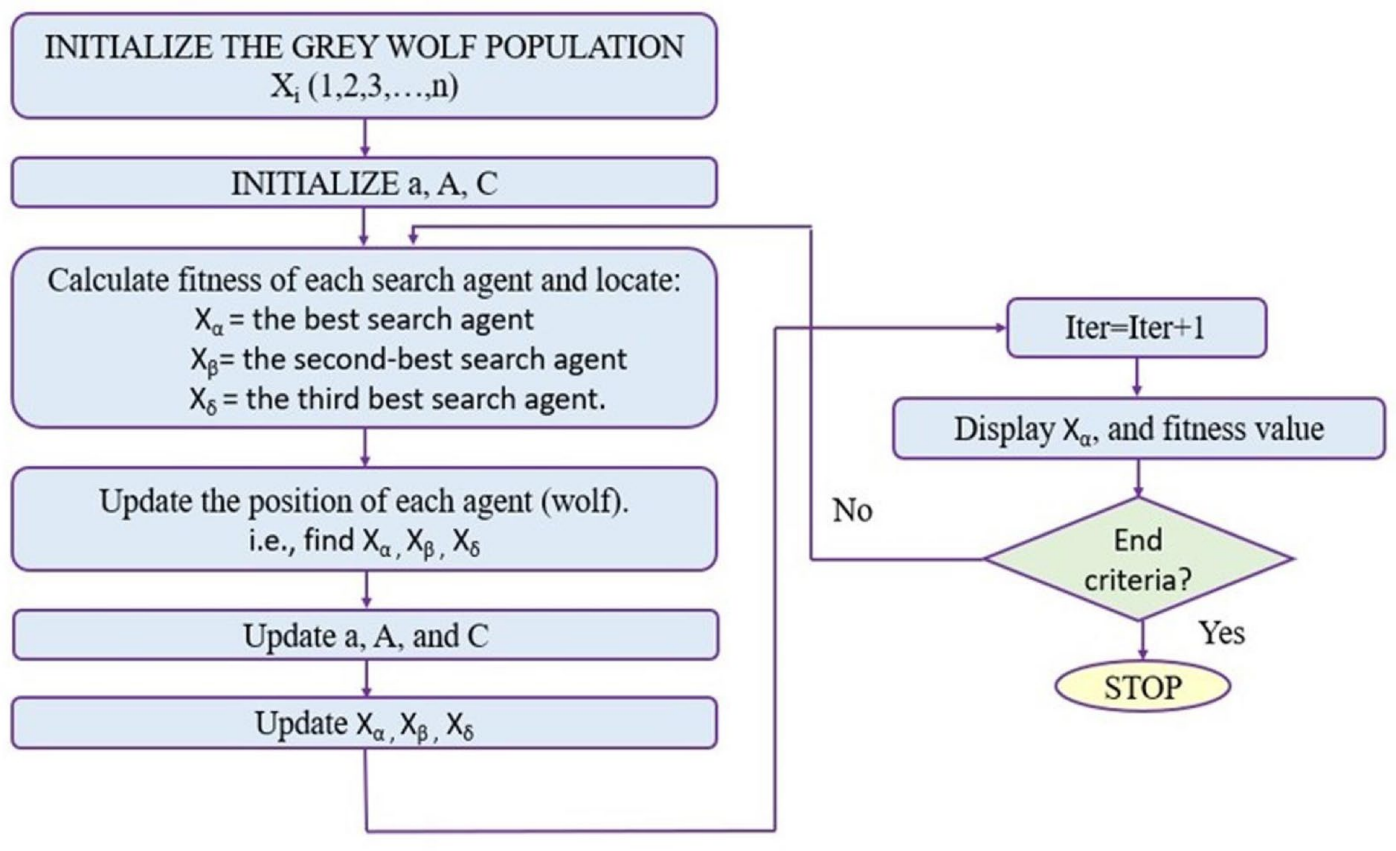
GWO algorithm flowchart^54^.

Figure 4 explains the algorithm of GWO. First, all agents’ initialization is done within the search space. The objective function is determined for all agents using the locations of the wolves. Then, the best position of GWO is calculated and updated in each iteration. Finally, as these steps are repeated, the alpha’s position, reflecting the optimal location, can be located.

## Proposed method

This section presents a detailed description of the proposed hybrid IPSO-GWO algorithm, which integrates Improved Particle Swarm optimization (IPSO) with Grey Wolf Optimization (GWO), inspired by the approach outlined in^45^. The IPSO-GWO algorithm begins with randomly generated particles, where each particle represents a potential solution to the workflow scheduling problem. The random solution is initially processed through the IPSO algorithm for half of the total iterations. The solution obtained after these iterations serves as the initial input for the GWO algorithm.

### Solution vector encoding (particle/agent representation)

**Fig. 5:**
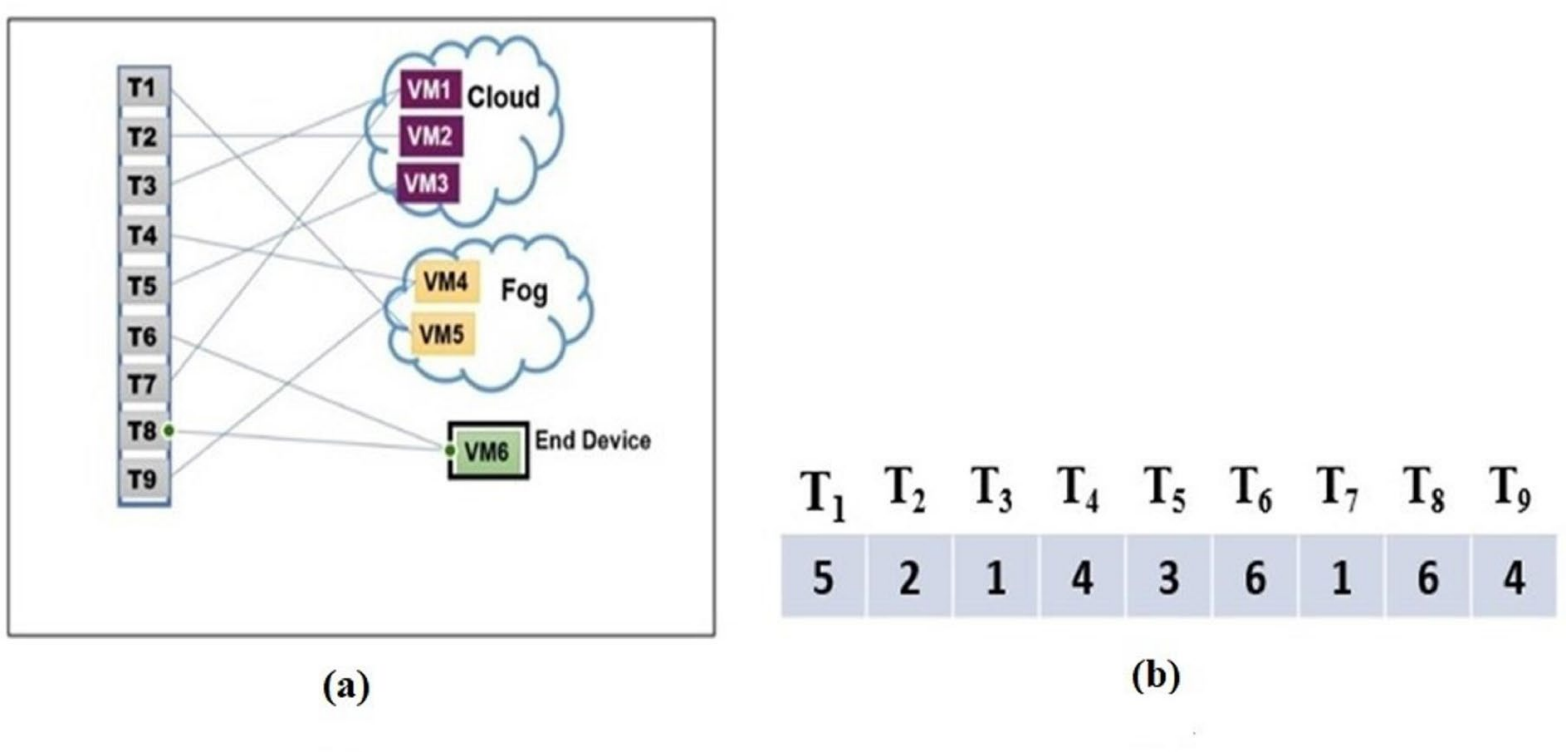
Scheduling process example: (**a**) Tasks/Virtual Machines mapping and (**b**) Solution vector example.

In IPSO-GWO, an individual represented by a particle for IPSO and wolves for GWO corresponds to a potential solution for task scheduling. (Particles / Wolves) are encoded using an M-dimensional array, where each of the M genes represents a task. The position of a gene in the array indicates the task’s sequence number, while its value is an integer k within the range [1, $\mathrm{V}\mathrm{M}\mathrm{s}$], where $\mathrm{V}\mathrm{M}\mathrm{s}$ is the total number of virtual machines. This value specifies that the respective task is assigned to node k. The K numbers are taken from the suitable VMs within the corresponding architecture layer. Figure 5 provides a graphical representation of a sample workflow consisting of 9 tasks assigned to a cloud-fog environment, which includes 3 cloud VMs, 2 fog VMs, and 1 VM for the ED. In this example, the particle is represented as P = (5, 2, 1, 4, 3, 6, 1, 6, 4), where each number denotes the specific VM to which a task is allocated. VM1 processes tasks {3, 7}, VM2 is assigned the task {2}, VM3 handles task {5}, the tasks {4, 9} are processed in VM4, the task {1} is assigned to VM5, while VM6 handles tasks {6,8}.

Virtual Machines (VMs) are deployed at end devices to provide a virtualized execution environment closer to the data source. Without VMs, end devices would only act as data generators or relays, sending all tasks upward to fog or cloud layers. By integrating lightweight VMs at the edge, their impact on the performance of the proposed technique is:

- End devices can execute small, latency-sensitive tasks locally, reducing reliance on higher layers.
- Virtualization allows for isolation and resource management, enabling multiple applications to run concurrently even on constrained devices.
- It supports scalability and flexibility, since tasks can be dynamically migrated between end devices, fog, and cloud depending on resource availability.

The proposed IPSO-GWO algorithm is based on a predefined number of iterations, which in this model has been set to 100. The solution population is initially composed of a set of random vectors representing potential solutions within the search space. As the iterations progress, the algorithm incrementally refines these vectors, aiming to converge on the optimal solution through the interplay of IPSO mechanisms and grey wolf behavior.

The IPSO-GWO algorithm operates in two phases, which are declared below:

1. Phase 1 - IPSO:
  Particles represent task-VM mappings.
  Each particle updates its velocity using Eq. (9) and position using Eq. (10).
  The inertia weight ($\mathrm{w}$) is decreased linearly from ${\mathrm{w}}_{\mathrm{b}\mathrm{e}\mathrm{g}\mathrm{i}\mathrm{n}}$ to ${\mathrm{w}}_{\mathrm{e}\mathrm{n}\mathrm{d}}$ across iterations to balance exploration and exploitation using Eq. (11).
  After $\mathrm{m}\mathrm{a}\mathrm{x}\mathrm{I}\mathrm{t}\mathrm{e}\mathrm{r}$/2, the best solution (global best) is passed to GWO.
2. Phase 2 - GWO:
  The best IPSO solution initializes the alpha wolf.
  Other wolves (solutions) are updated using the standard GWO encircling and hunting process.
  The final alpha wolf represents the optimal schedule (best map between tasks of workflow and virtual machines).

In the hybrid IPSO-GWO approach, the algorithm transitions at the midpoint of the total iterations. The global best particle obtained from IPSO is designated as the alpha wolf in GWO, while the remaining wolves are initialized by slightly perturbing the IPSO solutions—introducing minor variations around the best particle and randomly selected particles—to maintain diversity without compromising fitness. This strategy ensures that IPSO’s refined exploitation capabilities are retained, while GWO contributes enhanced exploration by dispersing wolves throughout the IPSO solution space. Importantly, this re-encoding preserves the original task-to-VM mappings, meaning the structure of the solution vector remains unchanged; only the representation shifts from “particle” to “wolf agent,” allowing fitness evaluation to remain consistent.

The hybrid IPSO-GWO achieves a balance between exploration and exploitation by sequentially applying the IPSO and the GWO algorithms.

- IPSO uses a linearly decreasing inertia weight, which starts high (favoring exploration) and decreases gradually (favoring exploitation in later iterations).
- After half the iterations, the refined solution from IPSO is passed as the initial alpha wolf to GWO, which enhances diversity (exploration) by updating wolf positions around multiple leaders (${\upalpha\:}$, ${\upbeta\:}$,${\updelta\:}$).
- This synergy ensures that IPSO refines solutions (exploitation), while GWO avoids local optima (exploration), striking a dynamic balance.

**Fig. 6:**
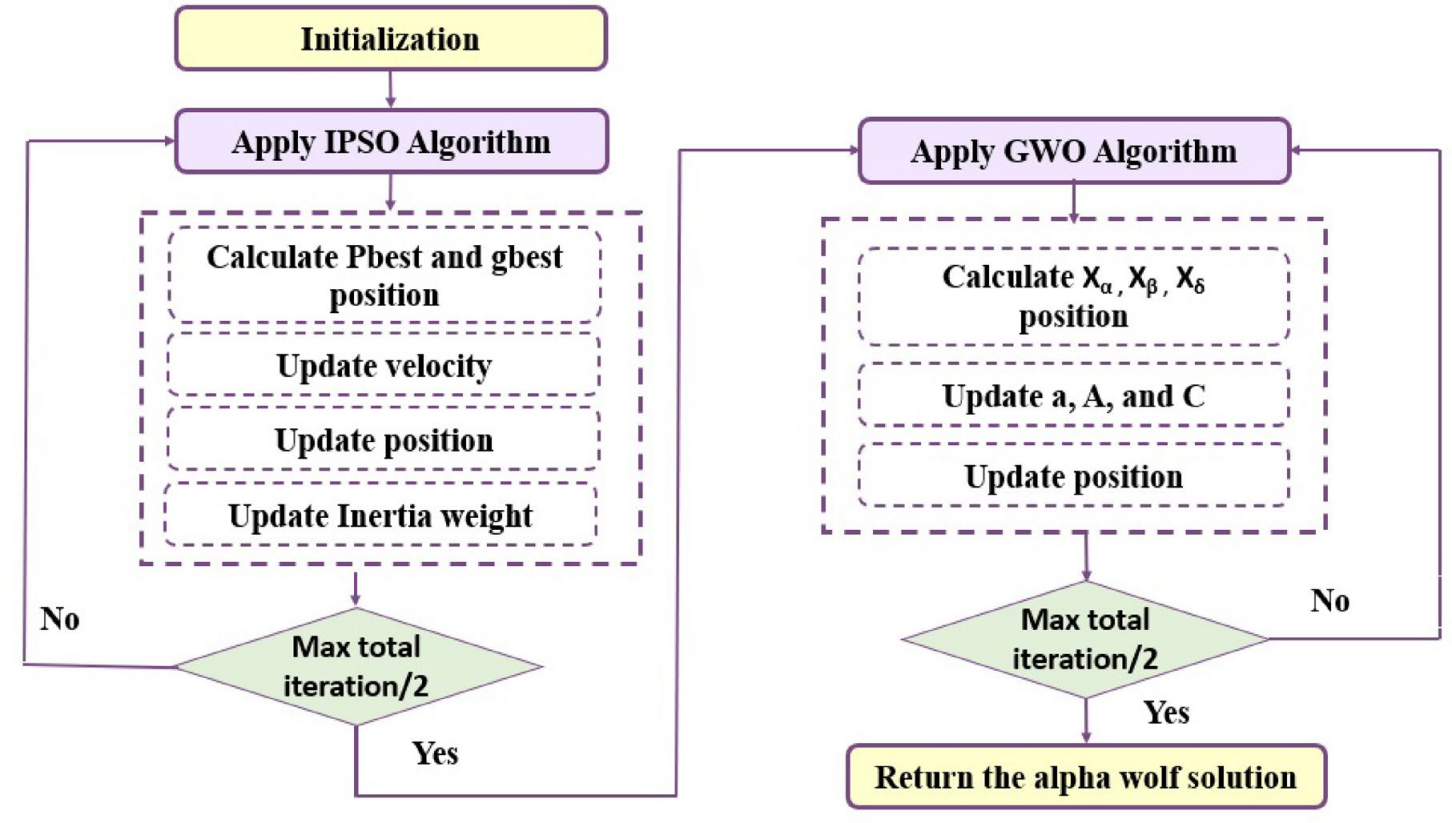
The IPSO-GWO algorithm flowchart.

Algorithm 1 outlines the IPSO-GWO algorithm, where the input includes the workflow (WF), $\mathrm{M}$ task, $\mathrm{m}\mathrm{a}\mathrm{x}\mathrm{I}\mathrm{t}\mathrm{e}\mathrm{r}$, $\mathrm{V}\mathrm{M}\mathrm{s}$ devices, and $\mathrm{p}\mathrm{o}\mathrm{p}\mathrm{S}\mathrm{i}\mathrm{z}\mathrm{e}$ for both IPSO equal particle number $\mathrm{N}$ and for GWO represent the number of wolves. It states in detail the IPSO-GWO steps. The IPSO-GWO flowchart is shown in Fig. 6.

**Algorithm 1:**
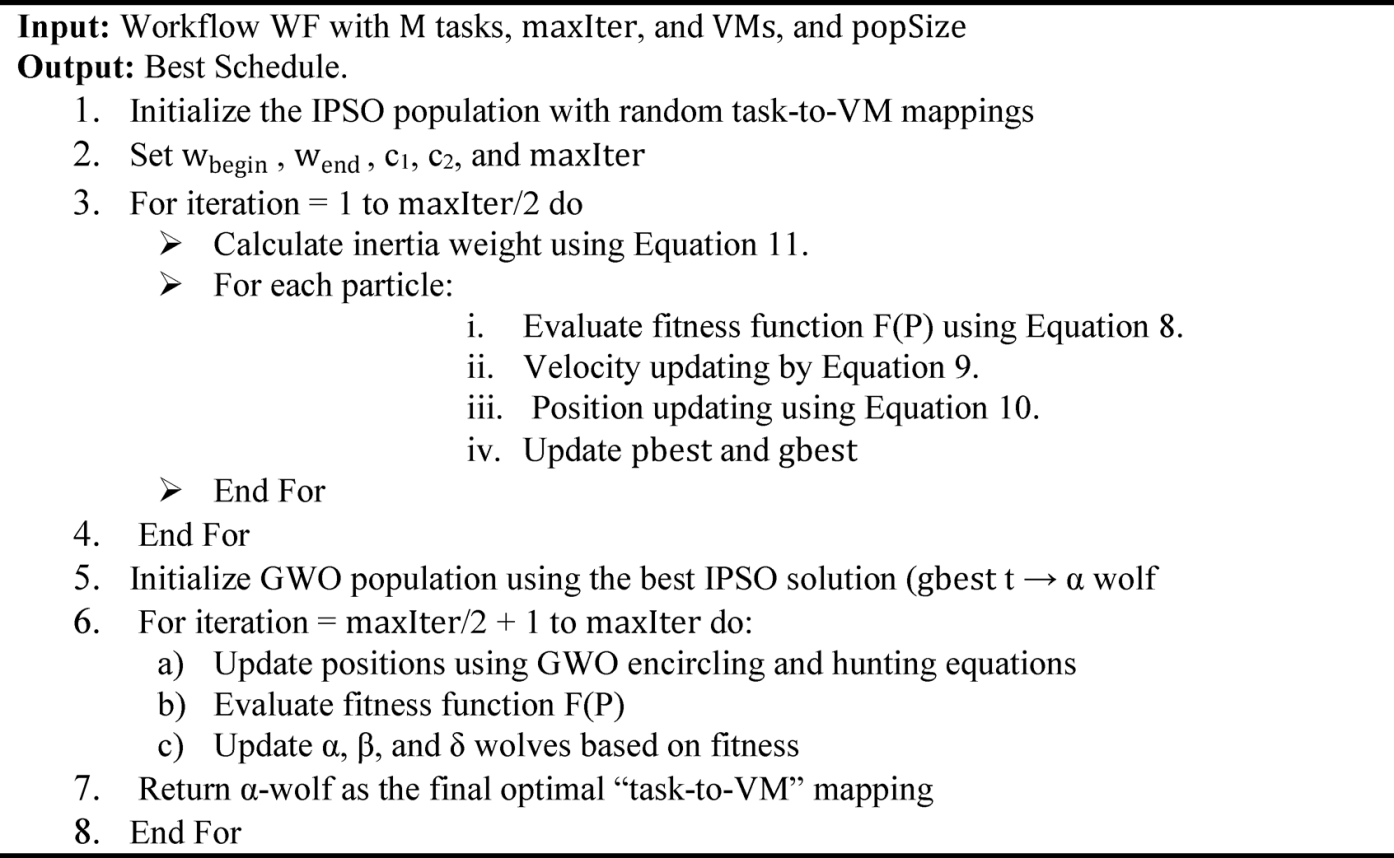
IPSO-GWO

## Evaluation of performance

### This work’s scientific workflow datasets

**Fig. 7:**
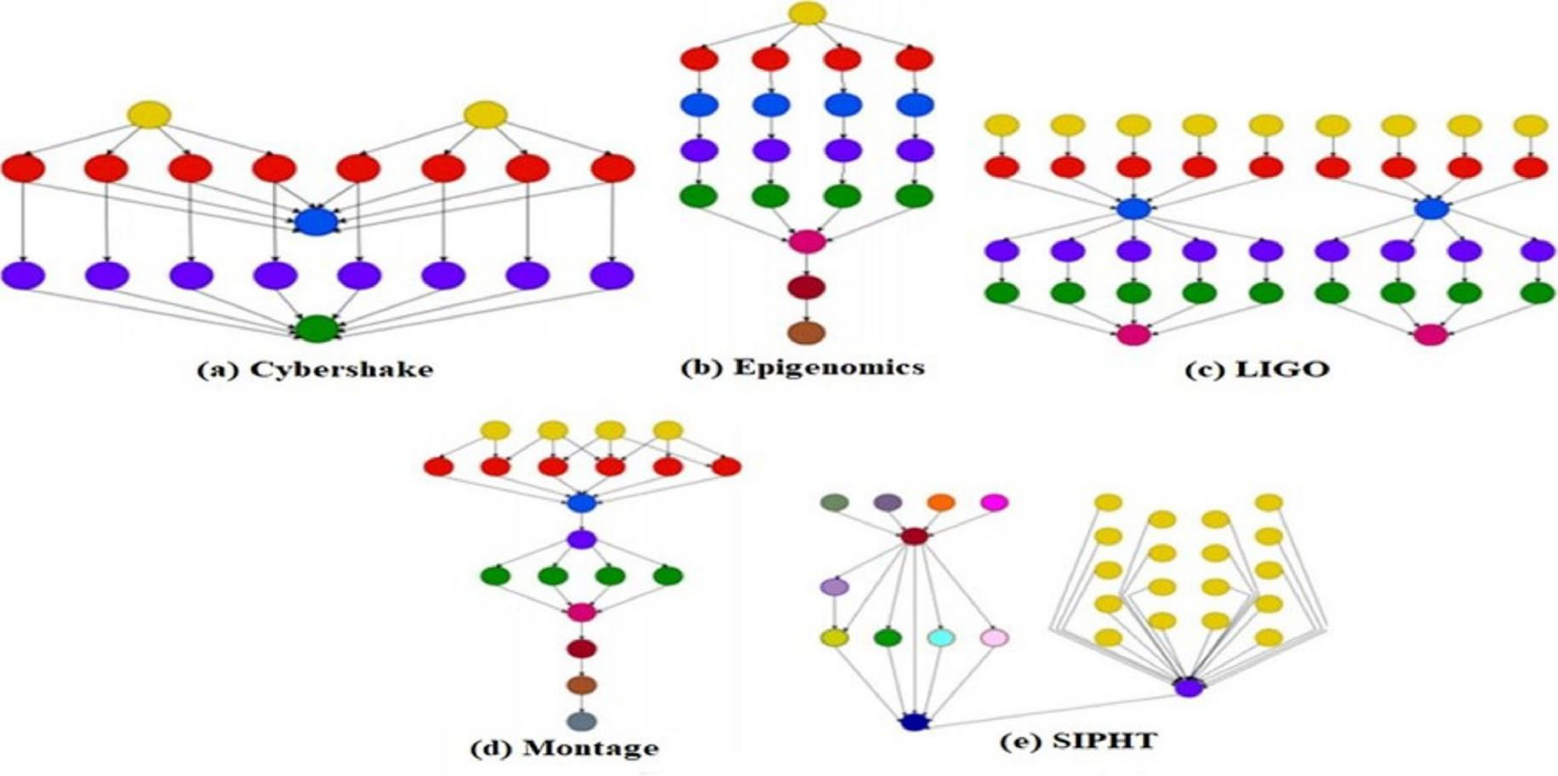
The architecture of Scientific workflows^17^.

This work uses all five workflow applications—Montage, Epigenomics, CyberShake, SIPHT, and LIGO—as benchmarks to assess performance and efficiency, characterized and defined in^56^ and introduced by the Pegasus framework^57^. Figure 7 presents a workflow diagram that visually represents all five scientific workflows used in this study. Montage workflow^58^ is a collection of tools developed by the NASA/IPAC Infrared Science Archive, designed to produce customized sky mosaics from input images in the Flexible Image Transmission System (FITS) format. The epigenomics workflow^59^ employs Pegasus to automate Illumina Solexa sequencing: data are split into blocks, mapped with Maq, cleaned of noise, aligned to the reference genome, and used to compute sequence density. The Laser Interferometer Gravitational-Wave Observatory (LIGO)^60^ aims to detect gravitational waves produced by various cosmic events. The LIGO Inspiral Analysis workflow is designed to process data from the coalescence of dense binary systems. The CyberShake workflow^61^ is a sophisticated computational workflow specifically designed for Probabilistic Seismic Hazard Analysis (PSHA). It is used to assess and characterize earthquake hazards in a specific region by simulating ground motions from potential earthquakes. SIPHT workflow^62^ is a bioinformatics workflow designed to automatically search and identify small Ribonucleic Acid (sRNA) encoding genes in bacterial genomes. Small RNAs (sRNAs) are short, non-coding RNA molecules that play critical roles in gene regulation, stress responses, and other cellular processes in bacteria. Identifying these sRNA genes is essential for understanding bacterial genomics and regulatory networks.

### This work’s simulation setting

The FogWorkflowSim simulator is used for emulating this work. It is executed and run using the Eclipse Java IDE. This work runs on a computer system outfitted with a 64-bit Windows 10 operating system, an Intel (R) Core (TM) i7-M 640 @ 2.80 GHz, and 6 GB of RAM. Each algorithm was repeated 100 times, and it was run with 25 particles.

The IPSO-GWO and baseline algorithms have been tested through simulations to reduce the sum of a weighted objective function. Table 2 outlines the parameter settings used in the simulation across different tiers of the IFC system. To evaluate the effectiveness of the proposed algorithm, each workflow is tested with three different task quantities. Table 3 presents the algorithm-specific parameter settings used for evaluation. All algorithms have been simulated using a weighted sum approach, considering makespan, energy consumption, and total cost as objective factors. The weighted coefficients are all set equally to 0.2. These workflows are provided with varying task amounts, such as 100, 500, and 1000, to analyze performance across different scales. To evaluate the average performance of the algorithms, simulations are conducted 100 times for different workflow types and task quantities.

### Simulation results for IPSO-GWO

The performance of the IPSO-GWO algorithm is evaluated against four other scheduling algorithms: PSO^17,29^, IPSO^31^, GWO^35^, and GSA^63^. For a fair comparison, all five algorithms are tested under the same simulated conditions. Each algorithm runs 100 times for each scientific workflow. The population size is identical across all algorithms and corresponds to the number of defined solutions. By maintaining the same size of the population, the evaluation provides a balanced assessment of performance across different algorithms. This detailed analysis highlights the relative strengths and weaknesses of each scheduling strategy. The parameters are set to match those of the baseline algorithms.

**Fig. 8:**
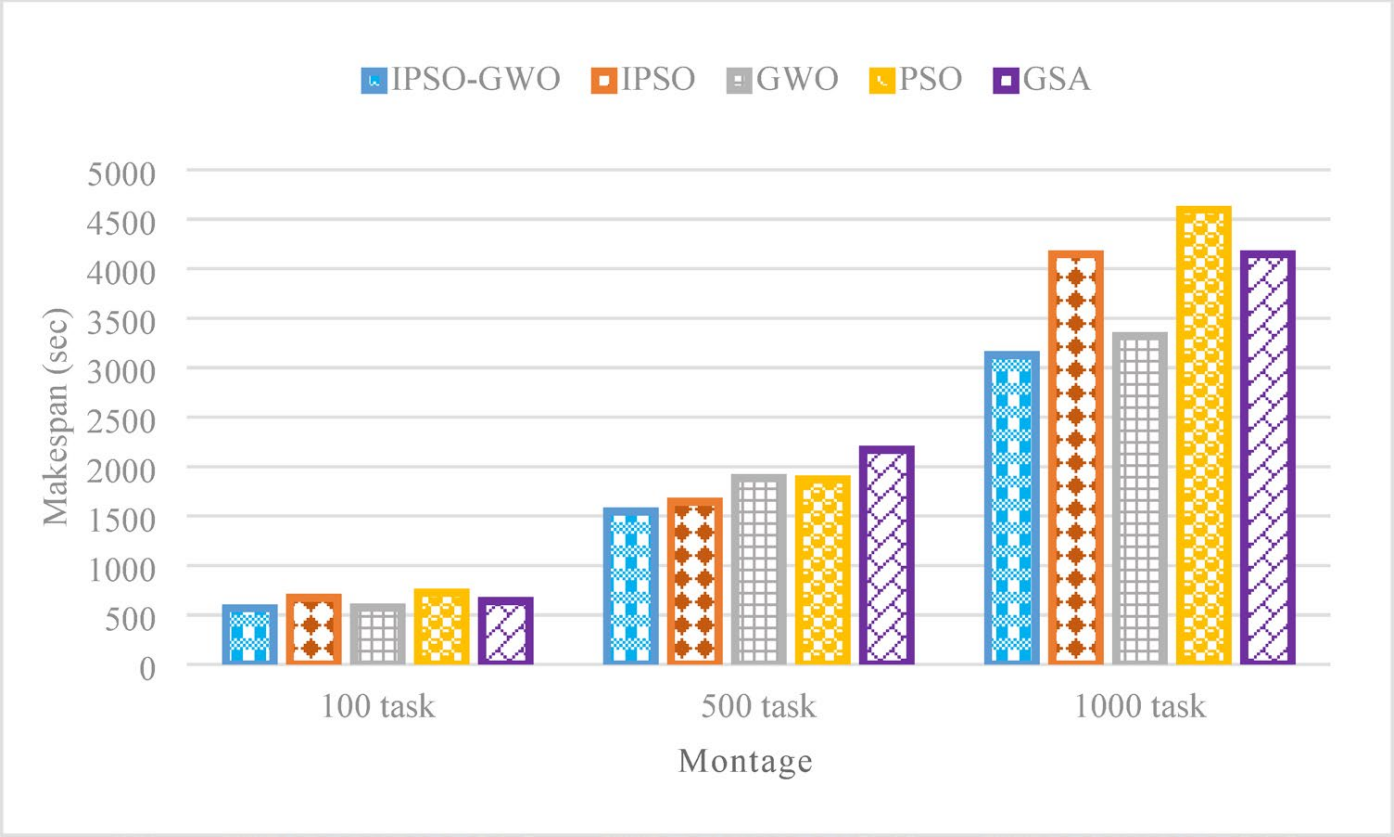
Makespan comparison in the Montage workflow.

**Fig. 9:**
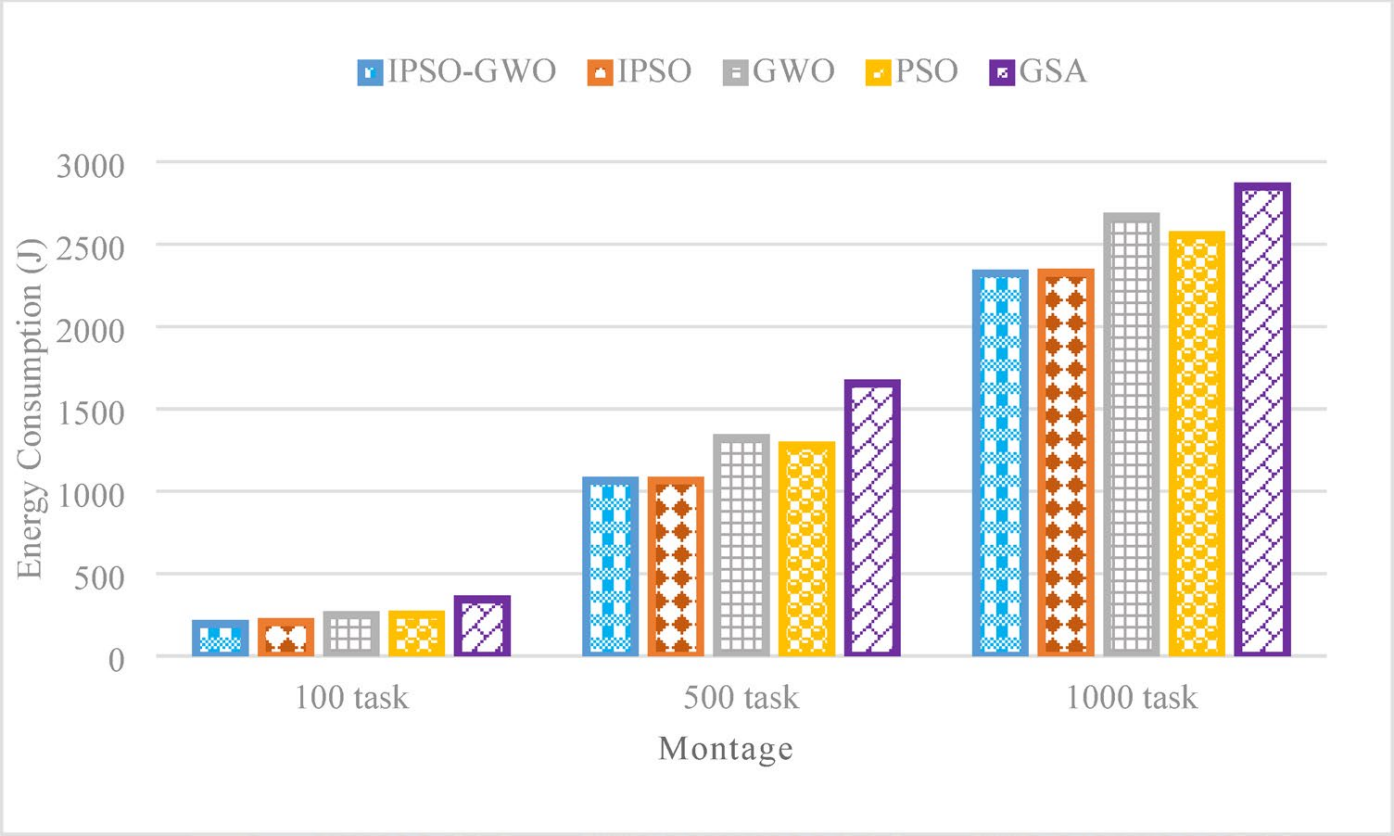
Energy consumption comparison in the Montage workflow.

**Fig. 10:**
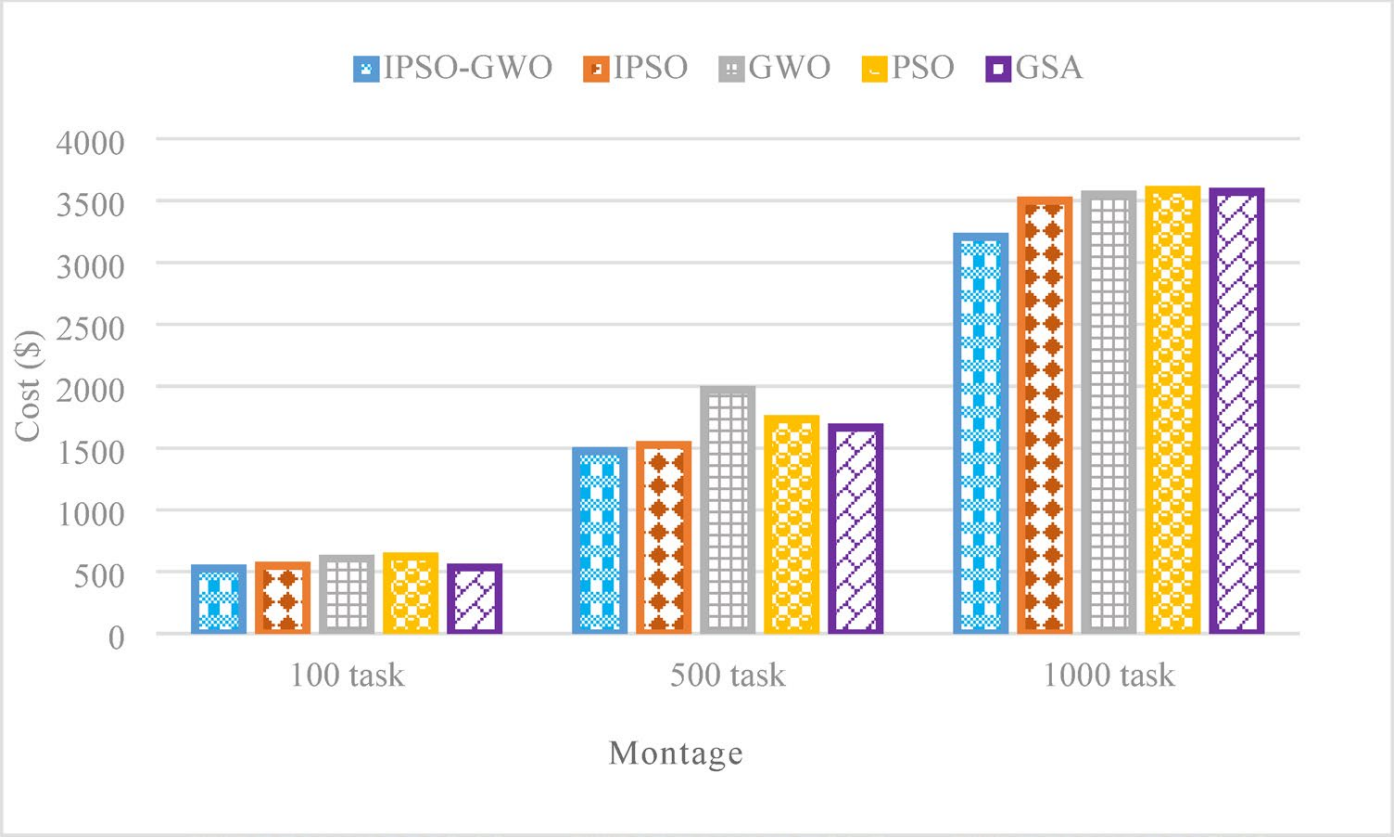
Total cost comparison in the Montage workflow.

Figures 8, 9 and 10 illustrate the performance of the algorithms using the Montage workflow with varying task sizes, specifically 100, 500, and 1000 tasks. The assessment considers three primary metrics: $\mathrm{M}\mathrm{S}$, $\mathrm{T}\mathrm{E}$, and $\mathrm{T}\mathrm{C}$. The suggested IPSO-GWO method consistently outperforms PSO in all test cases, achieving notable reductions in $\mathrm{M}\mathrm{S}$ by as much as 21.51%, 17.32%, and 32% for task sizes of 100, 500, and 1000, respectively, as shown in Fig. 8. Additionally, it reduces $\mathrm{M}\mathrm{S}$ by 15.68%, 6.04%, and 24.62% compared to IPSO, 0.99%, 17.89%, and 5.76% compared to GWO, and finally by 11.24%, 28.77%, and 24.62% compared to GSA.

**Table 2:** FogWorkflowSim environment parameter setting for IPSO-GWO^38^.

Parameters	EDs	FDs	CDs
Number of servers/devices	5	5	5
Processing rate (MIPS)	ED1-1000 ED2-1000 ED3-1000 ED4-1000 ED5-1000	FD1- 1200 FD2- 1300 FD3- 1400 FD4- 1500 FD5- 1600	CD1-1600 CD2-1700 CD3-1800 CD4-1900 CD5-2000
Task execution cost ($)	0	FD1- 0.1 FD2- 0.2 FD3- 0.3 FD4-0.4 FD5-0.5	CD1- 0.5 CD2- 0.6 CD3- 0.7 CD4-0.8 CD5-0.9
Communication cost ($)	0	0.01	0.02
Working power (MW)	700	800	1600
Idle power (MW)	30	40	1300
Uplink bandwidth (Mbps)	20	10	1
Downlink bandwidth (Mbps)	40	10	10

**Table 3:** Parameters of the used algorithms.

Algorithm	Parameters	Values
PSO	Number of particles (N)	25
	$\mathrm{C}1,\mathrm{C}2$	2
	$\mathrm{w}$	1
	$${\mathrm{r}}_{1},{\mathrm{r}}_{2}$$	[0,1]
IPSO	Number of particles (N)	25
	$\mathrm{C}1,\mathrm{C}2$	2
	${\mathrm{w}}_{\mathrm{b}\mathrm{e}\mathrm{g}\mathrm{i}\mathrm{n}}$	0.8
	${\mathrm{w}}_{\mathrm{e}\mathrm{n}\mathrm{d}}$	0.3
	$${\mathrm{r}}_{1},{\mathrm{r}}_{2}$$	[0,1]
GWO	Wolves number$\:\left(\mathrm{p}\mathrm{o}\mathrm{p}\mathrm{S}\mathrm{i}\mathrm{z}\mathrm{e}\right)$	25
	$$\mathrm{a}$$	[2,0]
	$\mathrm{A}$	0
	$\mathrm{C}$	0
	$${\mathrm{r}}_{1},{\mathrm{r}}_{2}$$	[0,1]
GSA	Agent number $\left(\mathrm{p}\mathrm{o}\mathrm{p}\mathrm{S}\mathrm{i}\mathrm{z}\mathrm{e}\right)$	25
	${\mathrm{G}}_{0}$	1
	${{\upalpha\:}}_{\mathrm{G}\mathrm{S}\mathrm{A}}$	20
	$${\mathrm{r}\mathrm{a}\mathrm{n}\mathrm{d}}_{\mathrm{j}}$$	[0,1]

The energy consumption of fog and cloud devices was calculated based on the model in^24,27,35,38^, using fixed parameters such as voltage and frequency. These parameters were kept constant to simplify the simulation and ensure fair comparability across the tested algorithms. In FogWorkflowSim, the energy profile of each device is defined directly through the idle power and busy power values specified when creating a fog device. Hence, the simulation does not dynamically model voltage and frequency scaling but instead relies on these predefined constant power parameters.

Figure 9 presents the simulation outcomes of the evaluated algorithms concerning $\mathrm{T}\mathrm{E}$. The proposed IPSO-GWO achieves a significant reduction in $\mathrm{T}\mathrm{E}$ compared to PSO, with decreases of 22.28%, 16.81%, and 9.10% for task sizes of 100, 500, and 1000, respectively. Furthermore, it decreases $\mathrm{T}\mathrm{E}$ by 5.50%, 0.16%, and 0.20% compared to IPSO, 21.50%, 19.55%, and 12.98% compared to GWO, and by 43.56%, 35.72%, and 18.51% compared to GSA.

Figure 10 demonstrates that the IPSO-GWO algorithm effectively reduces $\mathrm{T}\mathrm{C}$ across different task sizes. Specifically, it achieves reductions of 15.61%, 14.92%, and 10.64% compared to PSO for 100, 500, and 1000 tasks, respectively. Additionally, it lowers $\mathrm{T}\mathrm{C}$ by 4%, 3.36%, and 8.41% compared to IPSO, 13.42%, 25.15%, and 9.64% compared to GWO, 1.78%, 11.46%, and 10.21% compared to GSA.

**Fig. 11:**
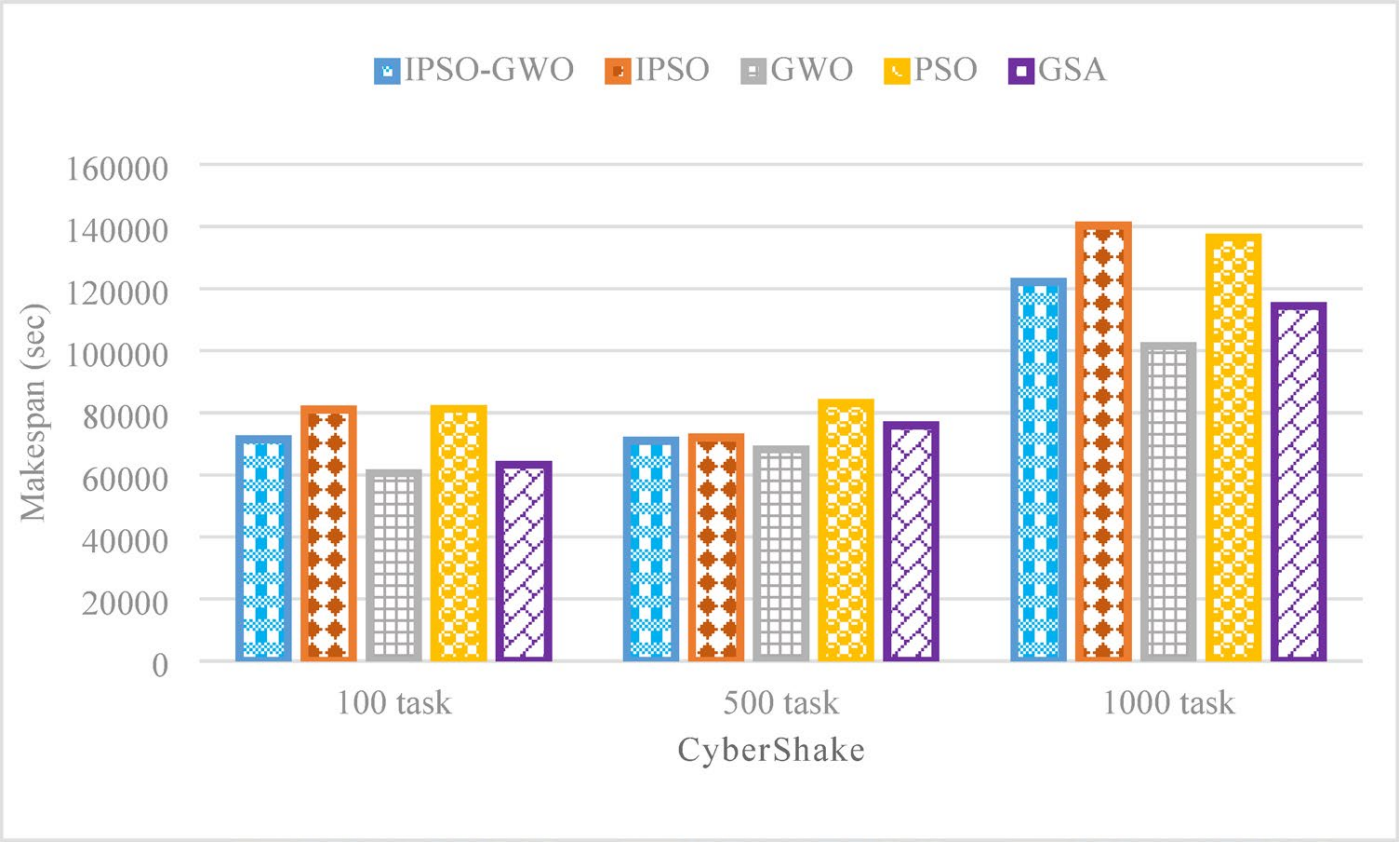
Makespan comparison in the CyberShake workflow.

**Fig. 12:**
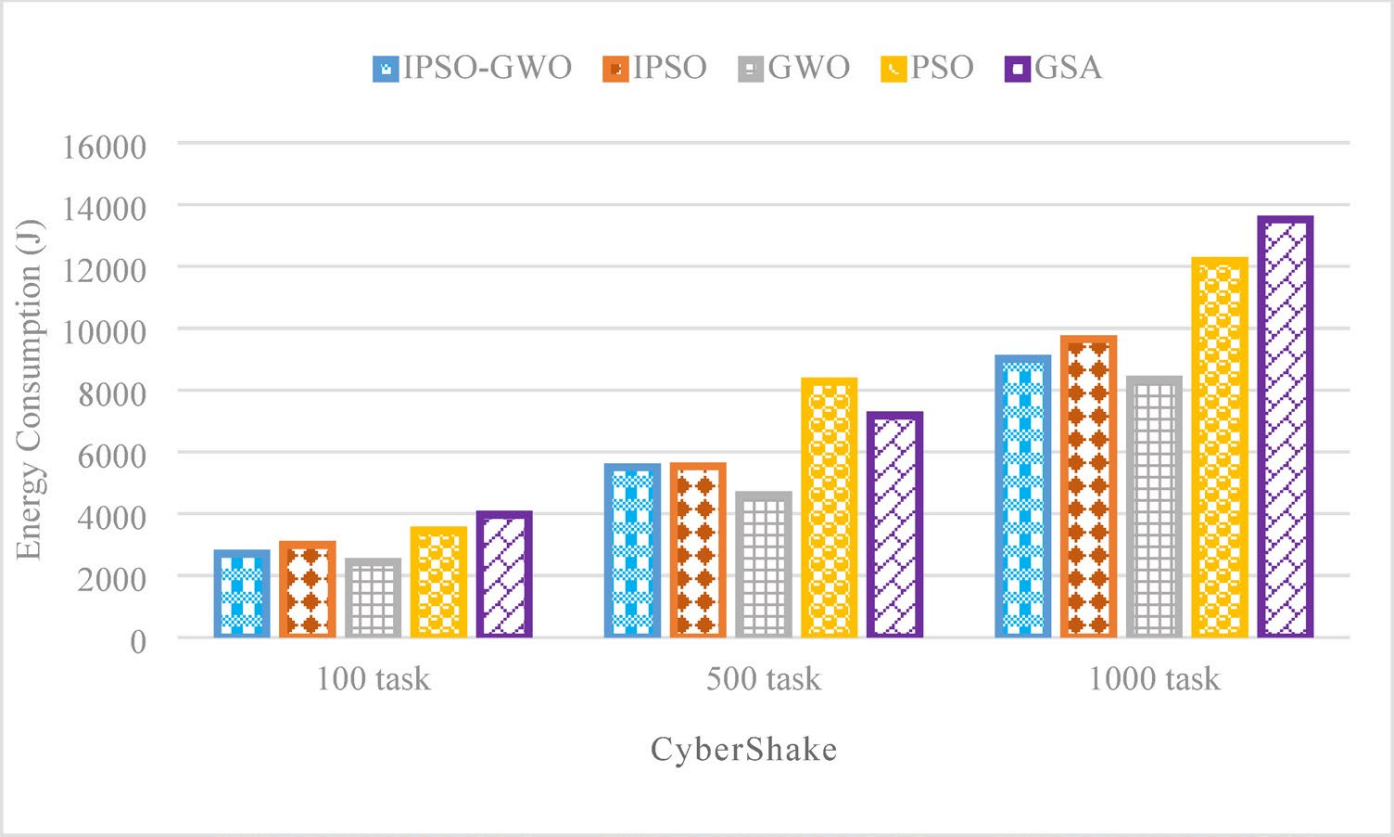
Energy consumption comparison in the CyberShake workflow.

**Fig. 13:**
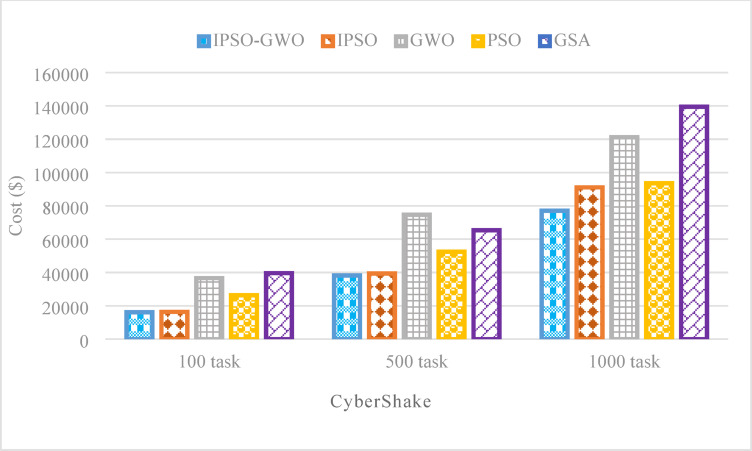
Total cost comparison in the CyberShake workflow.

Figures from Figs. 11, 12 and 13 present a performance evaluation of the algorithms when applied to the CyberShake workflow, analyzing different task sizes of 100, 500, and 1000. The comparison is based on three key performance indicators: $\mathrm{M}\mathrm{S}$, $\mathrm{T}\mathrm{E}$, and $\mathrm{T}\mathrm{C}$. The proposed IPSO-GWO doesn’t achieve superior performance regarding reduction $\mathrm{M}\mathrm{S}$ as shown in Fig. 11. It is better than PSO in reducing $\mathrm{M}\mathrm{S}$ by 12.05%, 14.64%, and 10.48% for 100, 500, and 1000 tasks, respectively. Additionally, it decreases $\mathrm{M}\mathrm{S}$when compared to IPSO by 11.83%, 1.43%, and 12.95%. But when compared to GWO, it increases $\mathrm{M}\mathrm{S}$ by 15.29%, 3.90%, and 16.90% for 100, 500, and 1000 tasks, respectively. Finally, it shows an increase in terms of $\mathrm{M}\mathrm{S}$ when compared to GSA by 11.49% and 6.32% for 100 and 1000 tasks, respectively, while it reduces $\mathrm{M}\mathrm{S}$ than GSA by -6.94% for using 500 tasks.

Figure 12 displays the simulation results for the CyberShake workflow of the analyzed algorithms concerning$\:\mathrm{T}\mathrm{E}$. The proposed IPSO-GWO outperforms PSO, IPSO, and GSA in minimizing $\mathrm{T}\mathrm{E}$. However, GWO surpasses IPSO-GWO in achieving the lowest $\mathrm{T}\mathrm{E}$. IPSO-GWO achieves a $\mathrm{T}\mathrm{E}$ reduction of 21.74%, 33.42%, and 25.99% compared to PSO for task sizes of 100, 500, and 1000, respectively. Similarly, it lowers $\mathrm{T}\mathrm{E}$ by 9.79%, 0.50%, and 6.56% when compared to IPSO and by 31.84%, 23.33%, and 33.34% relative to GSA. However, in comparison to GWO, it results in a $\mathrm{T}\mathrm{E}$ increase of 9.78%, 16.43%, and 7.49% for the same task sizes.

Figure 13 highlights the effectiveness of the IPSO-GWO algorithm in reducing $\mathrm{T}\mathrm{C}$ for various task sizes. The IPSO-GWO algorithm significantly reduces $\mathrm{T}\mathrm{C}$ compared to the baseline algorithms across different task sizes. For 100 tasks, $\mathrm{T}\mathrm{C}$ decreases by 1.34%, 55.92%, 38.85%, and 59.24% when compared to IPSO, GWO, PSO, and GSA, respectively. With 500 tasks, the reductions are 2.81%, 48.75%, 27.05%, and 41.43%, respectively. For 1000 tasks, $\mathrm{T}\mathrm{C}$ is lowered by 15.47%, 36.44%, 17.72%, and 44.79% in comparison to IPSO, GWO, PSO, and GSA.

**Fig. 14:**
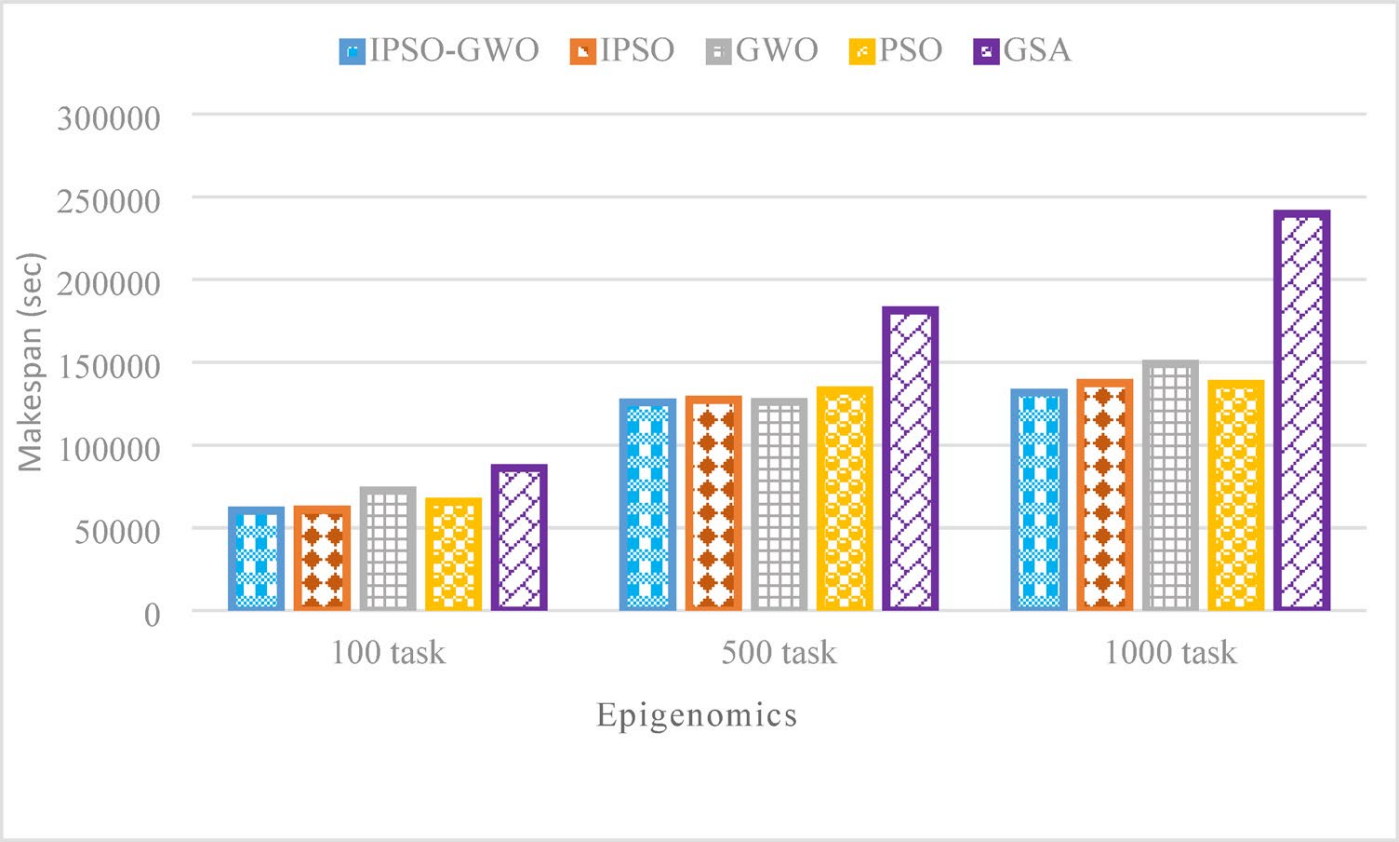
Makespan comparison in the Epigenomics workflow.

**Fig. 15:**
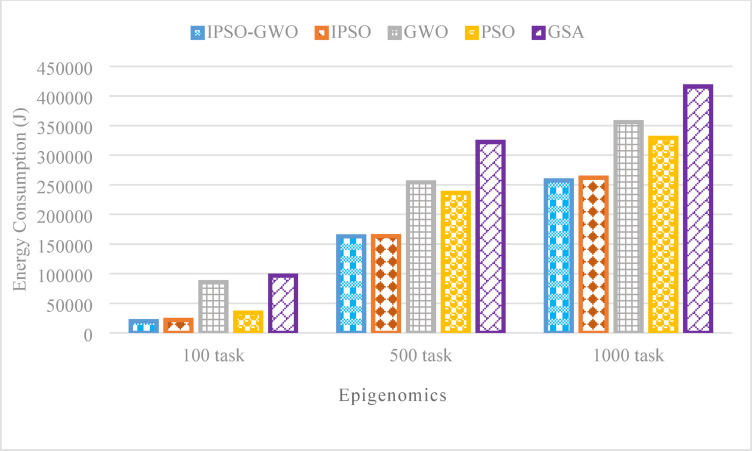
Energy consumption comparison in the Epigenomics workflow.

**Fig. 16:**
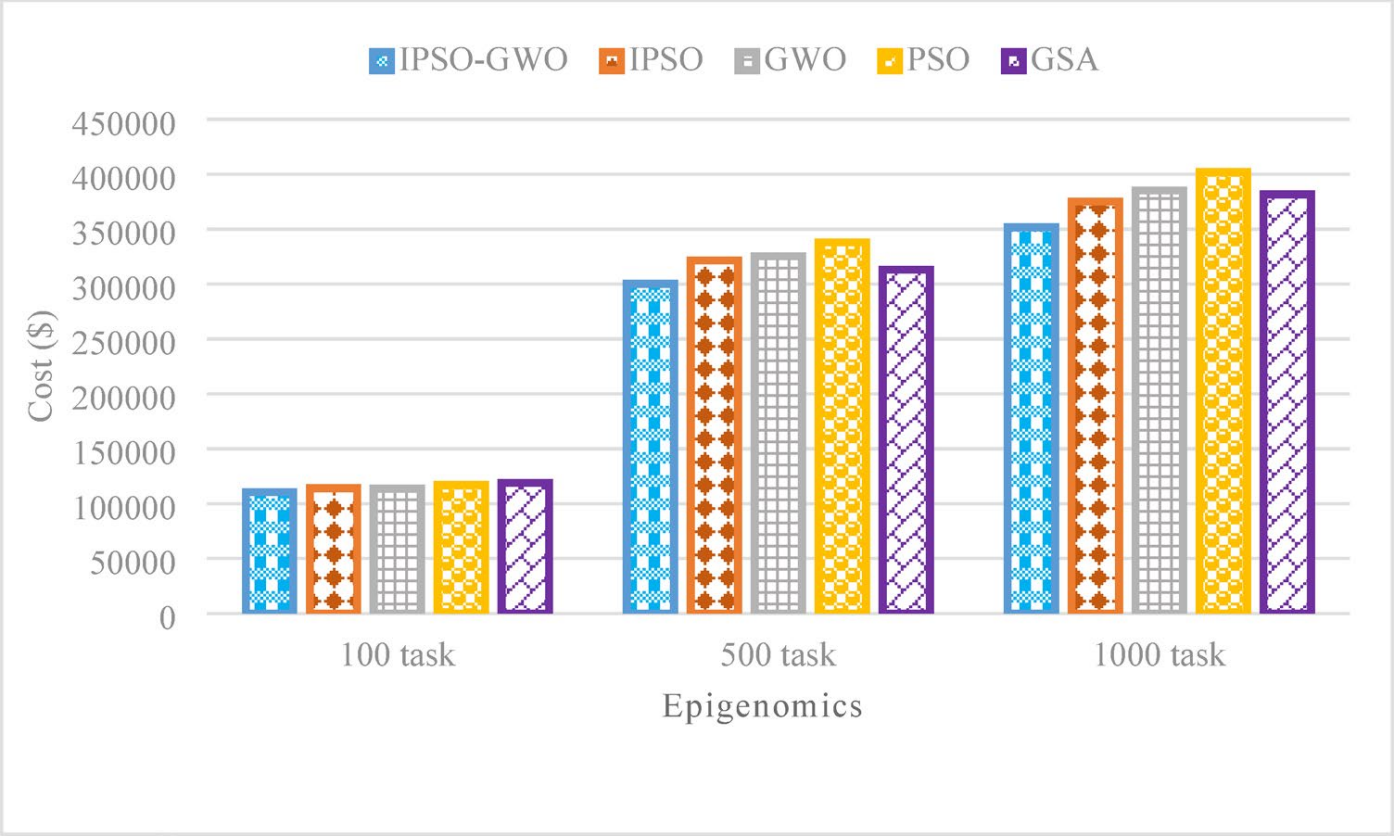
Total cost comparison in the Epigenomics workflow.

Figures 14, 15, and 16 showcase the performance analysis of the algorithms in the Epigenomics workflow, considering task sizes of 100, 500, and 1000. The evaluation focuses on three critical metrics: $\mathrm{M}\mathrm{S}$, $\mathrm{T}\mathrm{E}$, and $\mathrm{T}\mathrm{C}$. Figures show that IPSO-GWO excels all algorithms. Figure 14 illustrates that the proposed algorithm achieves a lower $\mathrm{M}\mathrm{S}$ compared to the baseline approaches. Specifically, it reduces $\mathrm{M}\mathrm{S}$ by 1.22%, 1.25%, and 4.22% compared to IPSO for task sizes of 100, 500, and 1000, respectively. When compared to GWO, the reductions are 16.89%, 0.17%, and 11.61%. Additionally, it decreases MS by 8.21%, 5.42%, and 3.88% relative to PSO, while achieving the most significant reduction against GSA, with improvements of 29.98%, 30.61%, and 45.06%.

Figure 15 declares that the proposed algorithm effectively minimizes $\mathrm{T}\mathrm{E}$ compared to the baseline methods. Specifically, it achieves reductions of 9.75%, 0.26%, and 1.79% over IPSO for task sizes of 100, 500, and 1000, respectively. Against GWO, the $\mathrm{T}\mathrm{E}$ decreases by 76.85%, 35.87%, and 27.68%. Similarly, it outperforms PSO with reductions of 42.13%, 31.03%, and 21.79%. The most substantial improvement is observed over GSA, with $\mathrm{T}\mathrm{E}$ lowered by 79.56%, 49.44%, and 38.12%.

Figure 16 highlights that the proposed algorithm significantly reduces $\mathrm{T}\mathrm{C}$ compared to the baseline approaches. It achieves $\mathrm{T}\mathrm{C}$ reductions of 3.22%, 6.60%, and 6.25% over IPSO for task sizes of 100, 500, and 1000, respectively. When compared to GWO, TE is lowered by 2.78%, 7.68%, and 8.70%. Additionally, the algorithm outperforms.

PSO with reductions of 5.55%, 11.16%, and 12.51%. Finally, it excels in minimizing $\mathrm{T}\mathrm{C}$ compared to GSA, achieving reductions of 7.06%, 4.07%, and 7.85% for task sizes of 100, 500, and 1000, respectively.

**Fig. 17:**
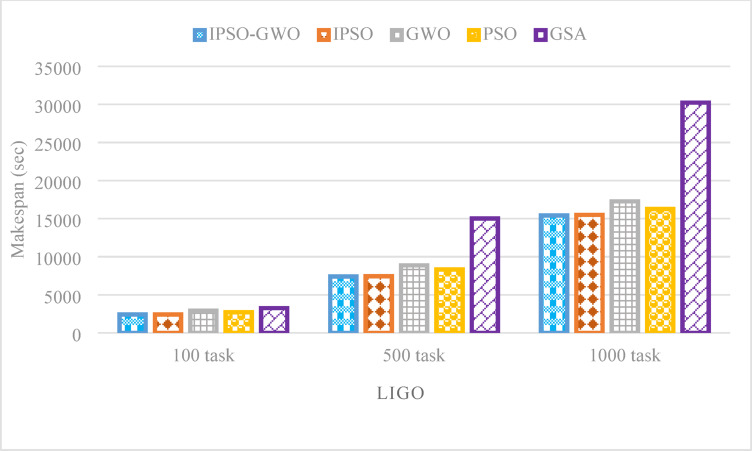
Makespan comparison in the LIGO workflow.

**Fig. 18:**
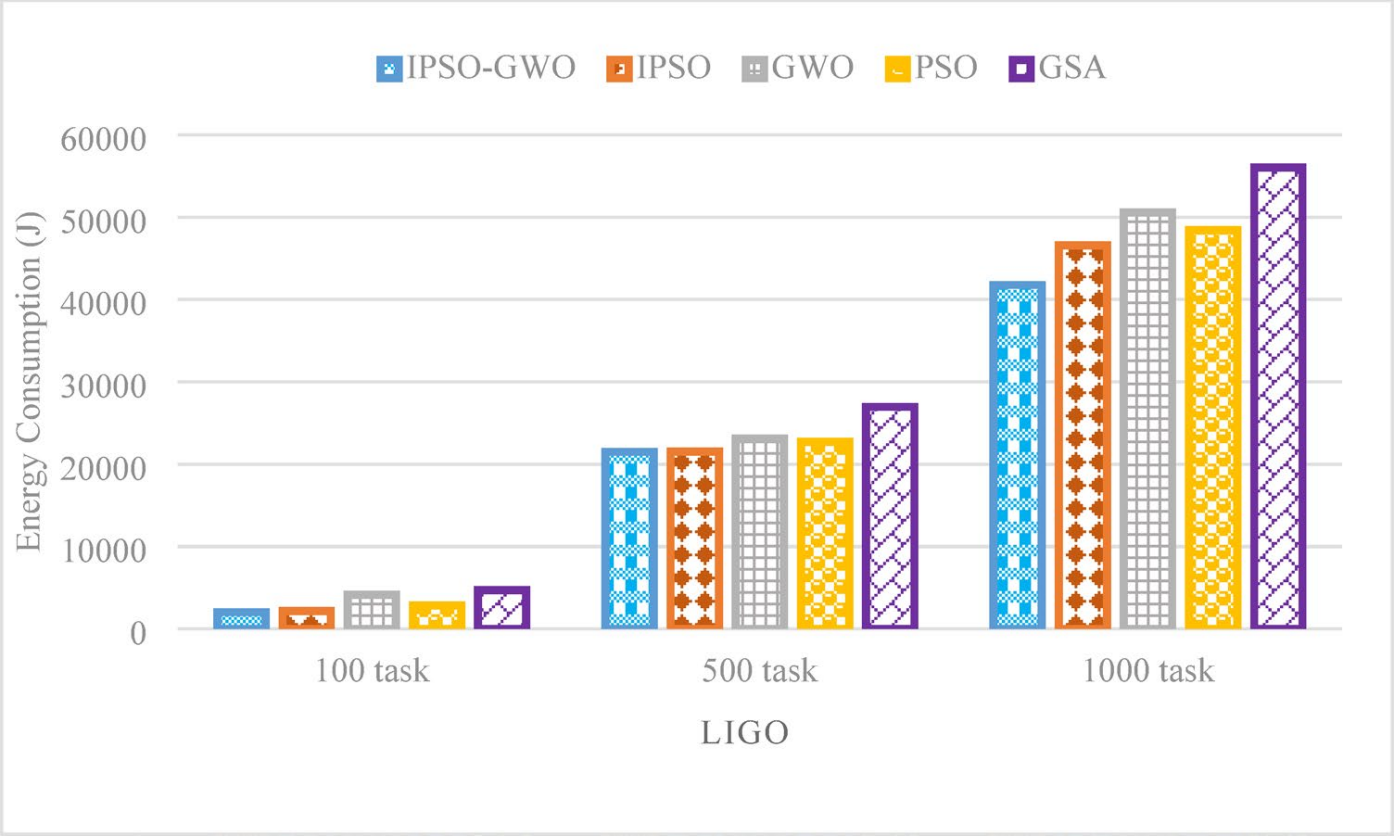
Energy consumption comparison in the LIGO workflow.

**Fig. 19:**
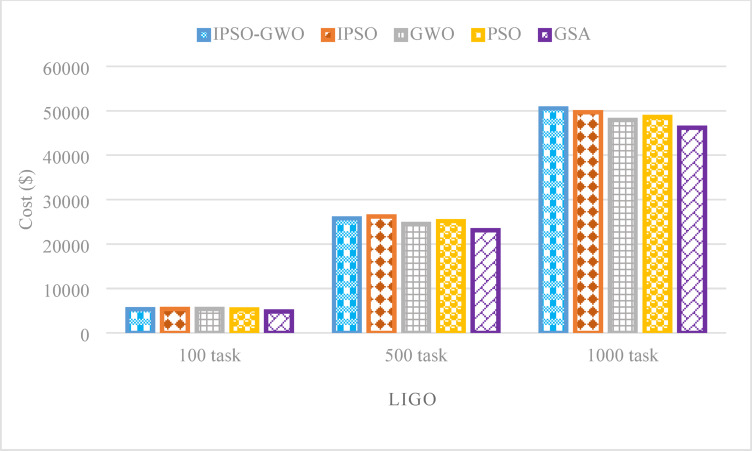
Total cost comparison in the LIGO workflow.

Figures 17, 18, and 19 illustrate the performance of the evaluated algorithms on the LIGO workflow with task sizes of 100, 500, and 1000. The assessment focuses on three key metrics: $\mathrm{M}\mathrm{S}$, $\mathrm{T}\mathrm{E}$, and $\mathrm{T}\mathrm{C}$. The proposed method consistently surpasses IPSO, achieving significant $\mathrm{M}\mathrm{S}$ reductions of 0.70%, 0.40%, and 0.55% for task sizes of 100, 500, and 1000, respectively, as depicted in Fig. 17. Additionally, it reduces $\mathrm{M}\mathrm{S}$ by 17.11%, 16.41%, and 10.78% compared to GWO, 11.31%, 11.01%, and 5.26% compared to PSO, 25.87%, 50.64%, and 49.01% compared to GSA.

Figure 18 presents the simulation results for $\mathrm{T}\mathrm{E}$, where IPSO-GWO demonstrates substantial improvements over the baseline algorithms. It achieves $\mathrm{T}\mathrm{E}$ reductions of 6.75%, 0.12%, and 10.31% compared to IPSO, while also decreasing $\mathrm{T}\mathrm{E}$ by 50.68%, 7.04%, and 17.42% compared to GWO, 28.91%, 5.44%, and 13.74% compared to PSO, and 56.87%, 20.28%, and 25.45% compared to GSA.

Figure 19 reveals that IPSO-GWO does not consistently achieve a lower total cost across all scenarios. While it reduces $\mathrm{T}\mathrm{C}$by 0.79% and 1.69% for task sizes of 100 and 500, respectively, compared to IPSO, resulting in a 1.73% increase in $\mathrm{T}\mathrm{C}$ for 1000 tasks. Additionally, IPSO-GWO reduces $\mathrm{T}\mathrm{C}$ only for 100 tasks when compared to GWO. However, for task sizes of 500 and 1000, it results in an increase of 5.16% and 5.50% in $\mathrm{T}\mathrm{C}$, respectively, compared to GWO. PSO outperforms IPSO-GWO in reducing $\mathrm{T}\mathrm{C}$, achieving improvements of 0.78%, 2.42%, and 4.03% for task sizes of 100, 500, and 1000, respectively. Additionally, GSA surpasses IPSO-GWO in minimizing $\mathrm{T}\mathrm{C}$, achieving reductions of 10.95%, 11.45%, and 9.45% for task sizes of 100, 500, and 1000, respectively. This highlights GSA’s effectiveness in cost optimization across different workload sizes.

**Fig. 20:**
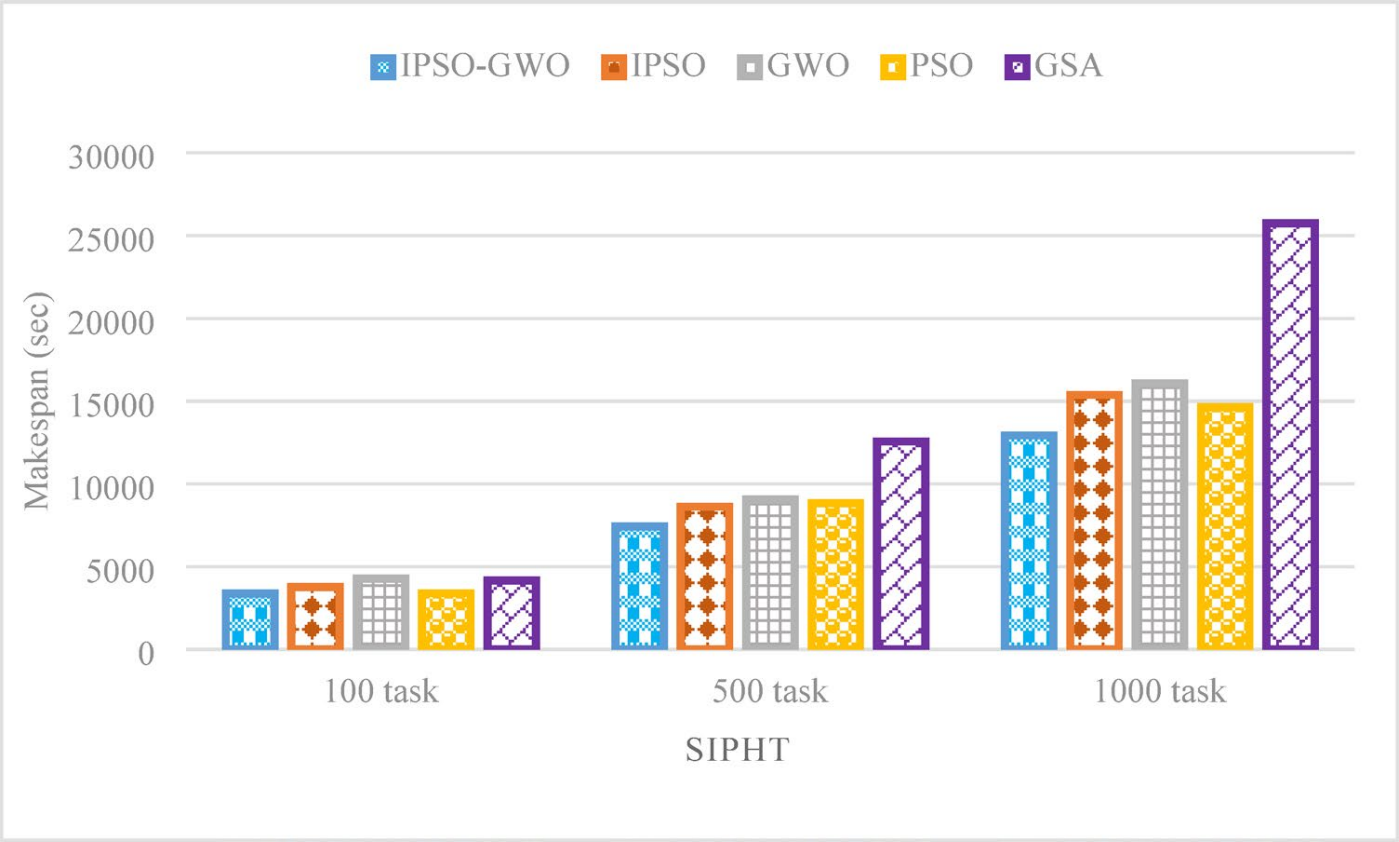
Makespan comparison in the SIPHT workflow.

**Fig. 21:**
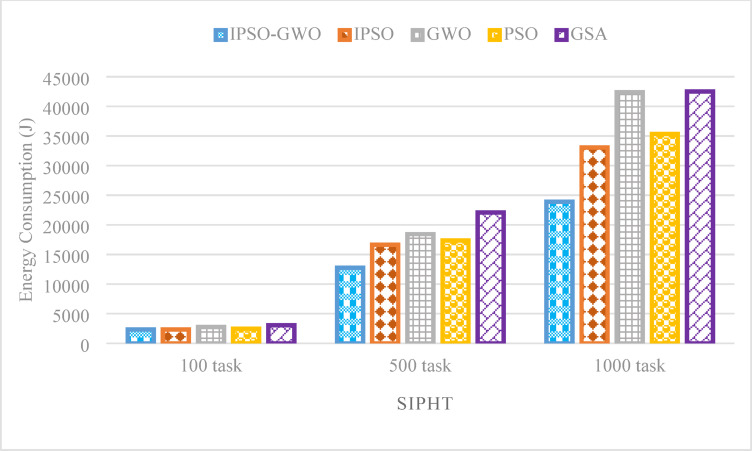
Energy consumption comparison in the SIPHT workflow.

**Fig. 22:**
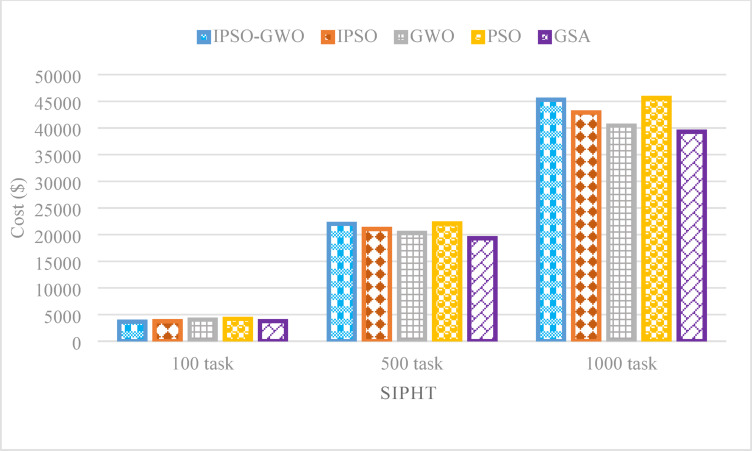
Total cost comparison in the SIPHT workflow.

Figures 20, 21 and 22 depict the performance evaluation of the tested algorithms on the SIPHT workflow for task sizes of 100, 500, and 1000. The analysis considers three critical performance indicators: $\mathrm{M}\mathrm{S}$, $\mathrm{T}\mathrm{E}$, and $\mathrm{T}\mathrm{C}$. These figures provide insights into how each algorithm performs in optimizing these metrics across varying task amounts, highlighting the strengths and weaknesses of each approach. The figures illustrate that IPSO-GWO outperforms all evaluated algorithms in minimizing $\mathrm{M}\mathrm{S}$ and $\mathrm{T}\mathrm{E}$ across different task sizes. However, it does not achieve the best performance in reducing $\mathrm{T}\mathrm{C}$, as other algorithms, such as GWO and GSA, demonstrate superior cost optimization.

Figure 20 underscores the superior performance of IPSO-GWO in minimizing $\mathrm{M}\mathrm{S}$ across various task sizes. Specifically, it achieves reductions of 10.33%, 13.93%, and 15.96% compared to IPSO for 100, 500, and 1000 tasks, respectively, demonstrating its effectiveness in optimizing makespan. Additionally, it outperforms GWO by reducing $\mathrm{M}\mathrm{S}$ by 20.86%, 17.98%, and 19.53% for the same task sizes, further reinforcing its efficiency. Compared to PSO, IPSO-GWO achieves reductions of 0.28%, 15.89%, and 11.63%, while showing the most significant improvement over GSA, lowering $\mathrm{M}\mathrm{S}$ by 18.81%, 40.89%, and 49.79%. These results highlight IPSO-GWO’s capability in effectively handling varying workloads and improving scheduling efficiency.

Figure 21 highlights that 1PSO-GWO significantly outperforms IPSO in minimizing $\mathrm{T}\mathrm{E}$, achieving reductions of 0.62%, 23.40%, and 27.71% for task sizes of 100, 500, and 1000, respectively. This demonstrates the effectiveness of IPSO-GWO in optimizing energy consumption across varying workload sizes. It further reduces $\mathrm{T}\mathrm{E}$ compared to GWO by 15.46%, 30.81%, and 43.61% for task sizes of 100, 500, and 1000, respectively. Additionally, IPSO-GWO achieves a reduction in $\mathrm{T}\mathrm{E}$ compared to PSO by 4.94%, 26.60%, and 32.36% for task sizes of 100, 500, and 1000, respectively. Finally, it demonstrates the most significant improvement over GSA, lowering $\mathrm{T}\mathrm{E}$ by 23.88%, 42.25%, and 43.79%, further emphasizing its efficiency in optimizing energy consumption.

Figure 22 declares that for 100 tasks, IPSO-GWO surpasses IPSO, GWO, PSO, and GSA in minimizing $\mathrm{T}\mathrm{C}$, achieving reductions of 2.63%, 9.46%, 12.72%, and 2.72%, respectively. However, for 500 and 1000 tasks, it increases $\mathrm{T}\mathrm{C}$ by 4.57% and 5.53% over IPSO, 8.44% and 11.96% over GWO, and 13.88% and 15.22% over GSA. It achieves a slight $\mathrm{T}\mathrm{C}$ reduction over PSO by 0.36% and 0.72%.

### Analysis of variance test (ANOVA)

ANOVA^64^ was applied to examine the statistical significance of the experimental results. This method assesses whether the means of multiple groups differ significantly, with the null hypothesis stating that all group means are equal, and the alternative hypothesis suggesting that at least one mean varies. It relies on statistical measures to evaluate the performance of the proposed algorithm and determine whether one outperforms the others significantly or if there are no meaningful differences. The primary objective of this study was to determine the most effective algorithm for solving the optimization problem, based on specific performance metrics: makespan, energy consumption, and cost. To do this, a thorough experimental evaluation was conducted, including 100 independent runs on both synthetic and real-world workflow datasets with varying task sizes. The performance was systematically compared across five different algorithms to ensure the robustness and reliability of the results. The outcomes of the experimental evaluation are summarized in Tables 4, 5, 6, 7 and 8, with each table presenting the results for the respective scenarios.

**Table 4:** ANOVA analysis for the montage workflow.

Task size	Metric	Mean value	F-statistic	*P*-value	Significant	Best algorithm
100	Makespan	568.52	0.70136	0.59512	No	IPSO-GWO
100	Energy consumption	194.01	19.024	3.2139e−09	Yes	IPSO-GWO
100	Cost	525.08	0.89501	0.47485	No	IPSO-GWO
500	Makespan	1545.9	1.3126	0.27977	No	IPSO-GWO
500	Energy consumption	1063.5	12.591	5.9047e−07	Yes	IPSO-GWO
500	Cost	1473.9	2.4072	0.063283	No	IPSO-GWO
1000	Makespan	1473.9	0.92321	0.45889	No	IPSO-GWO
1000	Energy consumption	2322.4	26.347	2.719e−11	Yes	IPSO-GWO
1000	Cost	3206.6	0.25994	0.90205	No	IPSO-GWO

The ANOVA test was conducted to evaluate the performance differences among IPSO-GWO, GWO, GSA, IPSO, and PSO for the Montage workflow across various task sizes (100, 500, and 1000 tasks) and three key metrics: Makespan, Energy Consumption, and Cost. For Makespan, the F-statistics and *P*-values indicate no statistically significant difference among the algorithms at any task size, although IPSO-GWO achieved the best average performance. For Energy Consumption, IPSO-GWO clearly outperformed the other algorithms, and ANOVA results showed highly significant differences (*P*-value < 0.001), confirming that the choice of algorithm strongly affects energy savings. For Cost, IPSO-GWO again had the best average, but the differences were not statistically significant. This is because the variation within each algorithm’s results was relatively large compared to the differences between algorithms. In other words, although IPSO-GWO has the lowest averages, the high internal variability prevents the differences from being statistically significant for certain metrics. Overall, IPSO-GWO demonstrates strong performance, especially in reducing energy consumption, while differences in Makespan and Cost are smaller and not statistically significant.

**Table 5:** ANOVA analysis for the cybershake workflow.

Task size	Metric	Mean value	F-statistic	*P*-value	Significant	Best algorithm
100	Makespan	60,529	0.43928	0.77953	No	GWO
100	Energy consumption	2438	1.3375	0.27078	No	GWO
100	Cost	16,148	1.8494	0.13601	No	IPSO-GWO
500	Makespan	68,202	2.202	0.083904	No	GWO
500	Energy consumption	4601.4	4.6731	0.0030643	Yes	GWO
500	Cost	38,266	1.5481	0.2046	No	IPSO-GWO
1000	Makespan	1.0152e + 05	2.1609	0.088771	No	GWO
1000	Energy consumption	8337.9	3.7288	0.010527	Yes	GWO
1000	Cost	77,035	2.3889	0.064892	No	IPSO-GWO

The ANOVA analysis for the CyberShake workflow demonstrates that performance differences among algorithms vary across task sizes and metrics. For small tasks (100), no statistically significant differences were observed in makespan, energy consumption, or cost. However, GWO achieved the lowest makespan and energy values, while IPSO-GWO provided the best cost efficiency. At 500 tasks, energy consumption showed significant differences (*p* = 0.003), with GWO emerging as the best performer, while IPSO-GWO maintained superior results in cost. For large-scale workloads (1000 tasks), energy consumption again displayed significant variation (*p* = 0.011), where GWO outperformed the other algorithms. Despite GWO’s dominance in energy optimization, IPSO-GWO consistently achieved the lowest execution cost across all task sizes, highlighting its effectiveness in cost-aware scheduling. These results confirm that the proposed IPSO-GWO algorithm strikes a strong balance between performance and cost efficiency, making it particularly suitable for scenarios where minimizing cost is a primary objective.

**Table 6:** ANOVA analysis for the epigenomics workflow.

Task size	Metric	Mean value	F-statistic	*P*-value	Significant	Best Algorithm
100	Makespan	60,310	13.336	1.8215e−07	Yes	IPSO-GWO
100	Energy consumption	19,783	67.533	1.532e−19	Yes	IPSO-GWO
100	Cost	98,880	3.9911	0.0069138	Yes	IPSO-GWO
500	Makespan	1.2586e + 05	20.464	1.1575e−09	Yes	IPSO-GWO
500	Energy consumption	1.6296e + 05	48.289	1.0053e−15	Yes	IPSO-GWO
500	Cost	3.031e + 05	6.2672	0.00042623	Yes	IPSO-GWO
1000	Makespan	1.3171e + 05	38.355	5.839e−14	Yes	IPSO-GWO
1000	Energy consumption	2.5733e + 05	58.944	2.5577e−17	Yes	IPSO-GWO
1000	Cost	3.5169e + 05	13.777	2.0576e−07	Yes	IPSO-GWO

The ANOVA analysis of the Epigenomics workflow highlights the strong performance of the IPSO-GWO algorithm across all task sizes and evaluation metrics. For a task size of 100, IPSO-GWO achieved the lowest makespan (mean = 60,310), energy consumption (mean = 19,783), and cost (mean = 98,880), with statistically significant differences confirmed by ANOVA (*p* < 0.01 in all cases). Similar trends were observed for larger task sizes. At 500 tasks, IPSO-GWO maintained superior results with reduced makespan, energy consumption (1.63 × 10⁵), and cost (3.03 × 10⁵), all supported by highly significant F-statistics (*p* ≤ 4.26 × 10⁻⁴). For the largest task size (1000), IPSO-GWO again dominated, recording a makespan of 1.32 × 10⁵, energy consumption of 2.57 × 10⁵, and cost of 3.52 × 10⁵, with strong statistical evidence (*p* ≤ 2.06 × 10⁻⁷). These findings confirm that IPSO-GWO consistently outperforms the compared algorithms, achieving statistically significant improvements in Makespan, energy efficiency, and cost-effectiveness across varying workflow scales.

**Table 7:** ANOVA analysis for the LIGO workflow.

Task size	Metric	Mean value	F-statistic	*P*-value	Significant	Best algorithm
100	Makespan	2412	5.76	0.00078624	Yes	IPSO-GWO
100	Energy consumption	2037.5	26.017	3.3033e−11	Yes	IPSO-GWO
100	Cost	4811.4	3.7023	0.010905	Yes	GSA
500	Makespan	7417.2	139.2	9.9655e−25	Yes	IPSO-GWO
500	Energy consumption	21,497	20.53	1.106e−09	Yes	IPSO-GWO
500	Cost	23,128	19.746	1.9154e−09	Yes	GSA
1000	Makespan	15,415	152	1.5959e−25	Yes	IPSO-GWO
1000	Energy consumption	41,764	35.354	2.3354e−13	Yes	IPSO-GWO
1000	Cost	46,178	13.579	2.4452e−07	Yes	GSA

The ANOVA test was conducted to evaluate the performance of IPSO-GWO, GWO, GSA, IPSO, and PSO for the LIGO workflow across different task sizes. The results demonstrate that IPSO-GWO achieved statistically significant improvements in both makespan and energy consumption across all task sizes (100, 500, and 1000). This indicates that the proposed IPSO-GWO algorithm consistently outperforms the comparative algorithms in terms of execution time efficiency and energy usage. However, for cost, the ANOVA results revealed that GSA was identified as the best-performing algorithm with statistically significant differences at all task sizes. These findings highlight the effectiveness of IPSO-GWO in optimizing time and energy, while also showing that cost optimization may favor alternative approaches such as GSA. Overall, IPSO-GWO proves to be the superior algorithm for critical performance metrics, while GSA demonstrates strength in cost-related outcomes.

**Table 8:** ANOVA analysis for the SIPHT workflow.

Task size	Metric	Mean value	F-statistic	*P*-value	Significant	Best algorithm
100	Makespan	3378.6	5.776	0.00077096	Yes	IPSO-GWO
100	Energy consumption	332.36	30.088	3.3342e−12	Yes	IPSO-GWO
100	Cost	3666	1.7881	0.14786	No	IPSO-GWO
500	Makespan	7423.7	35.005	2.7597e−13	Yes	IPSO-GWO
500	Energy consumption	12,752	14.946	7.6142e−08	Yes	IPSO-GWO
500	Cost	19,328	6.4179	0.00035629	Yes	GSA
1000	Makespan	12,926	45.886	2.517e−15	Yes	IPSO-GWO
1000	Energy consumption	23,901	31.451	1.6252e−12	Yes	IPSO-GWO
1000	Cost	39,308	15.704	4.0858e−08	Yes	GSA

The ANOVA analysis was performed to assess the comparative performance of IPSO-GWO, GWO, GSA, IPSO, and PSO on the SIPHT workflow with varying task sizes. The results indicate that IPSO-GWO consistently achieved statistically significant improvements in both makespan and energy consumption across all task sizes (100, 500, and 1000), highlighting its strong efficiency in reducing execution time and energy usage. For the cost metric, however, the results varied: while IPSO-GWO obtained the lowest mean value at smaller task sizes (100 tasks) without statistical significance, GSA emerged as the best-performing algorithm with statistically significant differences for larger task sizes (500 and 1000). These findings emphasize that the proposed IPSO-GWO algorithm is highly effective for optimizing critical performance factors such as time and energy, whereas GSA demonstrates a relative advantage in cost optimization. Overall, IPSO-GWO provides superior results in the most impactful performance dimensions of the SIPHT workflow.

The findings confirm that the proposed IPSO-GWO consistently outperforms the comparative algorithms in terms of makespan and energy consumption, achieving statistically significant improvements in most workflows, particularly Epigenomics, LIGO, and SIPHT, and in energy efficiency for Montage. While IPSO-GWO also attained the lowest average costs in Montage and CyberShake, the differences were not statistically significant due to high within-group variability. These outcomes highlight that the proposed IPSO-GWO is a robust and effective scheduling strategy, particularly excelling in reducing execution time and energy consumption, while also showing promising results in cost optimization.

### Complexity analysis

The computational complexity of the proposed IPSO-GWO scheduling algorithm is expressed in terms of four main variables: the population size ($\mathrm{p}\mathrm{o}\mathrm{p}\mathrm{S}\mathrm{i}\mathrm{z}\mathrm{e}$ number of candidate solutions), the maximum number of iterations ($\mathrm{m}\mathrm{a}\mathrm{x}\mathrm{I}\mathrm{t}\mathrm{e}\mathrm{r}$), the number of tasks in the workflow ($\mathrm{M}$), and the number of available processing nodes ($\mathrm{V}\mathrm{M}\mathrm{s}$, representing fog and cloud resources). In each iteration, the algorithms perform several operations whose costs are estimated separately. For PSO and IPSO, the velocity and position updates for each particle require O($\mathrm{M}$) operations, while fitness evaluation, which assigns tasks to VMs, requires O($\mathrm{M}$*$\mathrm{V}\mathrm{M}\mathrm{s}$). Summed across all particles, this leads to O($\mathrm{p}\mathrm{o}\mathrm{p}\mathrm{S}\mathrm{i}\mathrm{z}\mathrm{e}\mathrm{*}\mathrm{M}$*$\mathrm{V}\mathrm{M}\mathrm{s}$) per iteration and O($\mathrm{m}\mathrm{a}\mathrm{x}\mathrm{I}\mathrm{t}\mathrm{e}\mathrm{r}\mathrm{*}\mathrm{p}\mathrm{o}\mathrm{p}\mathrm{S}\mathrm{i}\mathrm{z}\mathrm{e}\mathrm{*}\mathrm{M}\mathrm{*}\mathrm{V}\mathrm{M}\mathrm{s}$) overall. GWO adds sorting operations of O($\mathrm{p}\mathrm{o}\mathrm{p}\mathrm{S}\mathrm{i}\mathrm{z}\mathrm{e}\:\mathrm{l}\mathrm{o}\mathrm{g}\:\mathrm{p}\mathrm{o}\mathrm{p}\mathrm{S}\mathrm{i}\mathrm{z}\mathrm{e}$), but this cost is negligible compared to fitness evaluation, so its overall complexity is also O($\mathrm{m}\mathrm{a}\mathrm{x}\mathrm{I}\mathrm{t}\mathrm{e}\mathrm{r}\mathrm{*}\mathrm{p}\mathrm{o}\mathrm{p}\mathrm{S}\mathrm{i}\mathrm{z}\mathrm{e}\mathrm{*}\mathrm{M}\mathrm{*}\mathrm{V}\mathrm{M}\mathrm{s}$). In contrast, GSA involves force computation among all pairs of agents, introducing a quadratic term O(${\mathrm{p}\mathrm{o}\mathrm{p}\mathrm{S}\mathrm{i}\mathrm{z}\mathrm{e}}^{2}\mathrm{*}\mathrm{M}$**)**, which makes its total complexity O($\mathrm{m}\mathrm{a}\mathrm{x}\mathrm{I}\mathrm{t}\mathrm{e}\mathrm{r}\mathrm{*}({\mathrm{p}\mathrm{o}\mathrm{p}\mathrm{S}\mathrm{i}\mathrm{z}\mathrm{e}}^{2}\mathrm{*}\mathrm{M}\:+\mathrm{p}\mathrm{o}\mathrm{p}\mathrm{S}\mathrm{i}\mathrm{z}\mathrm{e}\mathrm{*}\mathrm{M}\mathrm{*}\mathrm{V}\mathrm{M}\mathrm{s})$). Finally, the IPSO-GWO hybrid combines IPSO and GWO updates, both requiring O($\mathrm{p}\mathrm{o}\mathrm{p}\mathrm{S}\mathrm{i}\mathrm{z}\mathrm{e}\mathrm{*}\mathrm{M}$**)** alongside fitness evaluation, leading to the same overall complexity as PSO and GWO, i.e., O($\mathrm{m}\mathrm{a}\mathrm{x}\mathrm{I}\mathrm{t}\mathrm{e}\mathrm{r}\mathrm{*}\mathrm{p}\mathrm{o}\mathrm{p}\mathrm{S}\mathrm{i}\mathrm{z}\mathrm{e}\mathrm{*}\mathrm{M}\mathrm{*}\mathrm{V}\mathrm{M}\mathrm{s}$). This shows that IPSO-GWO preserves the computational efficiency of the lighter algorithms while avoiding the quadratic overhead of GSA.

### Limitations

While IPSO-GWO for task scheduling in a fog–cloud environment using a hybrid metaheuristic algorithm has its merits, there are also some limitations to consider. Here are a few limitations of IPSO-GWO:

1. Parameter Sensitivity: IPSO-GWO requires careful parameter tuning to achieve optimal performance, similar to other metaheuristic algorithms. Parameters such as population size, learning coefficients, and iteration limits significantly influence its efficiency. Identifying appropriate settings often demands extensive experimentation and domain expertise.
2. Convergence to Suboptimal Solutions: Like many swarm- and population-based algorithms, IPSO-GWO is not guaranteed to find the global optimum. Depending on the parameter configuration and problem complexity, it may converge prematurely to suboptimal solutions, particularly if the balance between exploration and exploitation is not properly maintained.
3. Limited Problem-Specific Adaptability: IPSO-GWO is a general-purpose optimization framework and does not inherently exploit problem-specific characteristics of fog–cloud task scheduling. This limitation may prevent it from fully leveraging domain knowledge or constraints, potentially leading to less efficient schedules in some scenarios.
4. Lack of Extensive Comparative Evaluation: Although IPSO-GWO shows promising results compared with baseline algorithms, its effectiveness has not yet been comprehensively benchmarked against a wider range of recent state-of-the-art hybrid approaches. Without such evaluations, it is difficult to fully assess its competitiveness, robustness, and adaptability across diverse workflow applications.
5. Implementation overhead: It arises from the switching mechanism between IPSO and GWO phases, which necessitates additional parameter tuning (e.g., inertia weight range, iteration ratio).

## Conclusion and future work

This section presents the key conclusions and offers recommendations for future research directions.

### Conclusion

This study presents a hybrid workflow scheduling algorithm, IPSO-GWO, designed to optimize scientific workflow execution in heterogeneous IoT–Fog–Cloud (IFC) environments. The proposed framework addresses multiple objectives, including makespan, total cost, and energy consumption in both active and idle states across all IFC layers, while explicitly considering the complexity of heterogeneous infrastructures. IPSO-GWO integrates Improved Particle Swarm Optimization (IPSO), which employs a linearly decreasing inertia weight to prevent premature convergence, with Grey Wolf Optimization (GWO) to balance global exploration and local exploitation.

Experimental evaluations using FogWorkflowSim demonstrate that IPSO-GWO outperforms PSO, IPSO, GWO, and hybrid GSA in minimizing makespan and energy consumption, achieving reductions of up to 50% and 70%, respectively. However, the algorithm does not consistently minimize total cost in high-load scenarios, reflecting a trade-off where faster and energy-efficient scheduling may involve higher-cost resources. Statistical analysis via ANOVA confirms the robustness of these findings. The hybrid approach maintains computational efficiency while avoiding the quadratic overhead associated with GSA, although it remains sensitive to parameter tuning and may converge to suboptimal solutions in some cases. Although recent hybrid algorithms such as HFSGA, SSKHOA, and AGWO have demonstrated promising performance in workflow scheduling, they were not included in the experimental comparison conducted in this study. The primary reason for their exclusion was the absence of publicly available implementations compatible with the FogWorkflowSim framework, as well as computational resource limitations that constrained extensive re-implementation and validation efforts. Instead, the proposed IPSO–GWO algorithm was compared against four widely recognized and reproducible baseline methods (PSO, IPSO, GWO, and GSA), ensuring fairness and reproducibility of results. Future research will focus on integrating these newer hybrid techniques into the same simulation environment to provide a more comprehensive performance assessment.

### Future work

Future research should explore integrating emerging optimization techniques, extending the framework to include objectives such as load balancing, security, and fault tolerance, and incorporating reinforcement learning to enhance adaptive, real-time task allocation. Overall, IPSO-GWO represents an effective and practical solution for energy- and cost-aware task scheduling in dynamic IFC environments, particularly for time- or energy-critical applications.

## Data Availability

The datasets used and/or analyzed during the current study are available from the corresponding author on reasonable request.

## References

[1] Atzori, L., Iera, A. & Morabito, G. The internet of things: A survey. *Comput. Netw.* **54** (15), 27872805. 10.1016/j.comnet.2010.05.010 (2010).

[2] Bhambri, P., Rani, S., Gupta, G. & Khang, A. *Cloud and Fog Computing Platforms for Internet of Things* (CRC, 2022).

[3] Hoseiny, F., Azizi, S. & Dabiri, S. Using the power of two choices for Real-Time task scheduling in Fog-Cloud computing. *2020 4th Int. Conf. Smart City Internet Things Appl. (SCIOT)* 18–23. 10.1109/SCIOT50840.2020.9250197 (2020).

[4] Bonomi, F., Milito, R., Zhu, J. & Addepalli, S. Fog computing and its role in the internet of things. In *Proceedings of the First Edition of the MCC Workshop on Mobile Cloud Computing*, 13–16. (2012). 10.1145/2342509.2342513

[5] Yousefpour, A. et al. All one needs to know about fog computing and related edge computing paradigms: A complete survey. *J. Syst. Architect.* **98**, 289–330. 10.1016/j.sysarc.2019.02.009 (2019).

[6] Rodriguez, M. A. & Buyya, R. A taxonomy and survey on scheduling algorithms for scientific workflows in IaaS cloud computing environments. *Concurr. Comput. Pract. Exp.*. **29** (8), e4041. 10.1002/cpe.4041 (2017).

[7] Jha, S., Lathrop, S., Nabrzyski, J. & Ramakrishnan, L. Incorporating scientific workflows in computing research processes. *Comput. Sci. Eng.* **21** (4), 4–6. 10.1109/MCSE.2019.2917987 (2019).

[8] Yu, J. & Buyya, R. A taxonomy of workflow management systems for grid computing arXiv:cs/0503025. arXiv. (2005). 10.48550/arXiv.cs/0503025

[9] Song, W. et al. Scientific workflow mining in clouds. *IEEE Trans. Parallel Distributed Syst.* **28**(10), 2979–2992. (2017). 10.1109/TPDS.2017.2696942

[10] Buyya, R., Pandey, S. & Vecchiola, C. Cloudbus Toolkit for market-oriented cloud computing. In (eds. Jaatun, M. G., Zhao, G. & Rong, C.) *Cloud Computing* 24–44 (Springer, 2009). 10.1007/978-3-642-10665-1_4

[11] Yakubu, I. Z. & Murali, M. An efficient meta-heuristic resource allocation with load balancing in IoT-Fog-cloud computing environment. *J. Ambient Intell. Humaniz. Comput.* **14** (3), 2981–2992. 10.1007/s12652-023-04544-6 (2023).

[12] Saif, F. A., Latip, R., Hanapi, Z. M. & Shafinah, K. Multi-Objective grey Wolf optimizer algorithm for task scheduling in Cloud-Fog computing. *IEEE Access.* **11**, 20635–20646. 10.1109/ACCESS.2023.3241240 (2023).

[13] Hafsi, H., Gharsellaoui, H. & Bouamama, S. Genetically-modified Multi-objective particle swarm optimization approach for high-performance computing workflow scheduling. *Appl. Soft Comput.* **122**, 108791. 10.1016/j.asoc.2022.108791 (2022).

[14] Singh, G. & Chaturvedi, A. K. Particle swarm optimization-based approaches for cloud-based task and workflow scheduling: A systematic literature review. In: *2021 2nd International Conference on Secure Cyber Computing and Communications (ICSCCC)* 350–358 (2021). 10.1109/ICSCCC51823.2021.9478149

[15] Kakkottakath Valappil Thekkepuryil, J., Suseelan, D. P. & Keerikkattil, P. M. An effective meta-heuristic based multi-objective hybrid optimization method for workflow scheduling in cloud computing environment. *Cluster Comput.* **24** (3), 2367–2384. 10.1007/s10586-021-03269-5 (2021).

[16] Farid, M., Latip, R., Hussin, M. & Abdul Hamid, N. A. W. A survey on QoS requirements based on particle swarm optimization scheduling techniques for workflow scheduling in cloud computing. *Symmetry* **12** (4). 10.3390/sym12040551 (2020).

[17] Subramoney, D. & Nyirenda, C. N. Multi-swarm PSO algorithm for static workflow scheduling in cloud-fog environments. *IEEE Access* **10**, 117199–117214 (2022). 10.1109/ACCESS.2022.3220239

[18] Wolpert, D. H. & Macready, W. G. No free lunch theorems for optimization. *IEEE Trans. Evol. Comput.* **1** (1), 67–82. 10.1109/4235.585893 (1997).

[19] Tang, J., Liu, G. & Pan, Q. A review on representative swarm intelligence algorithms for solving optimization problems: Applications and trends. *IEEE/CAA J. Automatica Sinica*. **8** (10), 1627–1643. 10.1109/JAS.2021.1004129 (2021).

[20] Črepinšek, M., Liu, S. H. & Mernik, M. Exploration and exploitation in evolutionary algorithms: A survey. *ACM Comput. Surveys*. **45** (3), 1–33. 10.1145/2480741.2480752 (2013).

[21] Verma, A. & Kaushal, S. A hybrid multi-objective particle swarm optimization for scientific workflow scheduling. *Parallel Comput.* **62**, 1–19. 10.1016/j.parco.2017.01.002 (2017).

[22] De Maio, V. & Kimovski, D. Multi-objective scheduling of extreme data scientific workflows in fog. *Future Generation Comput. Syst.* **106**, 171–184. 10.1016/j.future.2019.12.054 (2020).

[23] Nyirenda, C. N., Dawoud, D. S., Dong, F., Negnevitsky, M. & Hirota, K. A fuzzy multiobjective particle swarm optimized TS fuzzy logic congestion controller for wireless local area networks. *J. Adv. Comput. Intell. Intell. Inf.* **15** (1), 41–54. 10.20965/jaciii.2011.p0041 (2011).

[24] Yassa, S., Chelouah, R., Kadima, H. & Granado, B. Multi-objective approach for energy-aware workflow scheduling in cloud computing environments. *Sci. World J.* **2013**(1), 350934. 10.1155/2013/350934 (2013). 10.1155/2013/350934 PMC3835373 24319361

[25] Saif, F. A., Latip, R., Hanapi, Z. M. & Shafinah, K. Multi-objective grey wolf optimizer algorithm for task scheduling in cloud-fog computing. *IEEE Access* **11**, 20635–20646 (2023). 10.1109/ACCESS.2023.3241240

[26] Marler, R. T. & Arora, J. S. The weighted sum method for multi-objective optimization: New insights. *Struct. Multidisciplinary Optim.* **41** (6), 853–862. 10.1007/s00158-009-0460-7 (2010).

[27] Liu, X. et al. FogWorkflowSim: An automated simulation toolkit for workflow performance evaluation in fog computing. In: *2019 34th IEEE/ACM Int. Conf. Automated Softw. Eng. (ASE)*. **1114** (1117). 10.1109/ASE.2019.00115 (2019).

[28] Gamal, M., Awad, S., Abdel-Kader, R. F. & Elsalam, K. A. Efficient offloading and task scheduling in internet of thingth-cloud-fog environment. *Int. J. Electr. Comput. Eng. (IJECE)* **14** (4), 4 10.11591/ijece.v14i4.pp4445-4455 (2024).

[29] Subramoney, D. & Nyirenda, C. (n.d.). PSO-based workflow scheduling: A comparative evaluation of cloud and cloud-fog environments.

[30] Awad, S., Gamal, M., Salam, A. E., Abdel-Kader, R. F. & K., & Enhanced particle swarm optimization for task offloading and scheduling in Cloud-Fog environment. *Int. J. Telecommun.* **04** (02), 1–16. 10.21608/ijt.2024.308144.1060 (2024).

[31] Badr, S. A., Gamal, M., Ali, K. A. E. & Abdel-Kader, R. F. F. Multi-objective improved particle swarm optimization for efficient offloading algorithm in Fog-Cloud collaboration. *Suez Canal Eng. Energy Environ. Sci.* **2** (2), 17–26. 10.21608/sceee.2024.305699.1034 (2024).

[32] Varshney, S. & Srivastava, G. M. S. Efficient Workflow Scheduling in Fog-Cloud Environments using Ant Colony Optimization. In *2024 Second International Conference on Data Science and Information System (ICDSIS)* 1–5 (2024). 10.1109/ICDSIS61070.2024.10594408

[33] Hussain, M. & Begh, G. R. A multi-objective priority aware task scheduling in Fog–Cloud environment using improved meta-heuristic algorithm. *Res. Square*. 10.21203/rs.3.rs-3901654/v1 (2024).

[34] Medishetti, S. K., Karri, G. R. & Donthi, R. K. IBOA: Cost-aware task scheduling model for integrated cloud–fog environments. *Int. J. Inform. Technol. Comput. Sci.* **16** (5), 52 (2023).

[35] Soni, D. & Kumar, N. GWO-Based Workflow Scheduling in Cloud-Fog Environments. In *2023 International Conference on Electrical, Electronics, Communication and Computers (ELEXCOM)* 1–6 (2023). 10.1109/ELEXCOM58812.2023.10370034

[36] Ahmed, Y. N., Mohideen, S. P. & Pasha, M. Task scheduling in cloud-fog computing using discrete binary particle swarm meta-heuristic with modified sigmoid function. *J. Inform. Optim. Sci.* **44** (6), 1023–1033. 10.47974/JIOS-1226 (2023).

[37] Shukla, P. & Pandey, S. MAA: Multi-objective artificial algae algorithm for workflow scheduling in heterogeneous Fog-Cloud environment. *Res. Square*. 10.21203/rs.3.rs-1871192/v1 (2022).

[38] Shukla, P. & Pandey, S. DE-GWO: A multi-objective workflow scheduling algorithm for heterogeneous fog-cloud environment. *Res. Square* (2022). 10.21203/rs.3.rs-2141972/v1

[39] Bansal, S. & Aggarwal, H. A hybrid particle Whale optimization algorithm with application to workflow scheduling in cloud–fog environment. *Decis. Analytics J.* **9**, 100361. 10.1016/j.dajour.2023.100361 (2023).

[40] Bezdan, T. et al. Multi-objective task scheduling in cloud computing environment by hybridized Bat algorithm. *J. Intell. Fuzzy Syst.* **42** (1), 411–423. 10.3233/JIFS-219200 (2021).

[41] Singh, G. & Chaturvedi, A. K. Hybrid modified particle swarm optimization with genetic algorithm (GA) based workflow scheduling in cloud-fog environment for multi-objective optimization. *Cluster Comput.* **27** (2), 1947–1964. 10.1007/s10586-023-04071-1 (2024).

[42] Kumar, M. S., Reddy, K. G. & Donthi, R. K. (eds) (n.d.). SSKHOA: Hybrid metaheuristic algorithm for resource aware task scheduling in cloud-fog computing. *Int. J. Inf. Technol. Comput. Sci.* **16**(1), 1.

[43] Hussain, S. M. & Begh, G. R. Hybrid heuristic algorithm for cost-efficient QoS aware task scheduling in fog–cloud environment. *J. Comput. Sci.* **64**, 101828. 10.1016/j.jocs.2022.101828 (2022).

[44] Medishetty, S. K. & K, G. R. AGWO: Cost aware task scheduling in cloud fog environment using hybrid metaheuristic algorithm. *Int. J. Exp. Res. Rev.* **33**, 41–56. 10.52756/ijerr.2023.v33spl.005 (2023).

[45] Arora, N. & Kumar, R. HPSOGWO: A hybrid algorithm for scientific workflow scheduling in cloud computing. *Int. J. Adv. Comput. Sci. Appl.*10.14569/IJACSA.2020.0111078 (2020).

[46] Khaledian, N., Razzaghzadeh, S., Moazzami, S. & Kivi, P. N. TM-MOAOA: A two-stage task scheduling approach using TOPSIS and multi-objective Archimedes optimization in fog-cloud environment. *Computing* **107**, 155. 10.1007/s00607-025-01513-z (2025).

[47] Khaledian, N., Razzaghzadeh, S., Haghbayan, Z. & Völp, M. Hybrid Markov chain-based dynamic scheduling to improve load balancing performance in fog-cloud environment. *Sustain. Comput.: Inf. Syst.* **45**, 101077. 10.1016/j.suscom.2024.101077 (2025).

[48] Ghorbani, M., Khaledian, N. & Moazzami, S. ALBLA: An adaptive load balancing approach in edge-cloud networks utilizing learning automata. *Computing* **107**, 34. 10.1007/s00607-024-01380-0 (2024).

[49] Shahmirzadi, D., Khaledian, N. & Rahmani, A. M. Analyzing the impact of various parameters on job scheduling in the Google cluster dataset. *Cluster Comput.* **27**, 7673–7687. 10.1007/s10586-024-04377-8 (2024).

[50] Khaledian, N., Khamforoosh, K., Akraminejad, R., Abualigah, L. & Javaheri, D. An energy-efficient and deadline-aware workflow scheduling algorithm in the fog and cloud environment. *Computing* **106**, 109–137. 10.1007/s00607-023-01215-4 (2024).

[51] Khaledian, N., Khamforoosh, K., Azizi, S. & Maihami, V. IKH-EFT: An improved method of workflow scheduling using the Krill herd algorithm in the fog-cloud environment. *Sustain. Comput. Inf. Syst.* **37**, 100834. 10.1016/j.suscom.2022.100834 (2023).

[52] Krämer, M., Würz, H. M. & Altenhofen, C. Executing Cyclic scientific workflows in the cloud. *J. Cloud Comput.* **10** (1), 25. 10.1186/s13677-021-00229-7 (2021).

[53] Shami, T. M. et al. Particle swarm optimization: A comprehensive survey. *IEEE Access.* **10**, 10031–10061. 10.1109/ACCESS.2022.3142859 (2022).

[54] Mirjalili, S., Mirjalili, S. M. & Lewis, A. Grey Wolf optimizer. *Adv. Eng. Softw.* **69**, 46–61. 10.1016/j.advengsoft.2013.12.007 (2014).

[55] Alsamarai, N. A. & Uçan, O. N. Improved performance and cost algorithm for scheduling IoT tasks in Fog–Cloud environment using Gray Wolf optimization algorithm. *Appl. Sci.* **14** (4), 4 10.3390/app14041670 (2024).

[56] Bharathi, S. et al. Characterization of scientific workflows. 2008 third workshop on workflows in support of Large-Scale science, 1–10. (2008). 10.1109/WORKS.2008.4723958

[57] (N.d.). The Pegasus Website. Retrieved 30 January. from (2025). https://pegasus.isi.edu/

[58] Berriman, G. B. et al. Montage: A grid-enabled engine for delivering custom science-grade mosaics on demand. *Optimizing Sci. Return. Astronomy Through Inform. Technol.* **5493**, 221–232. 10.1117/12.550551 (2004).

[59] USC Epigenome Center – Departments Directory. (n.d.). Retrieved February 10. from (2025). https://departmentsdirectory.usc.edu/usc-epigenome-center/

[60] LIGO Lab | Caltech | MIT. (n.d.). LIGO Lab | Caltech. Retrieved February 10. from (2025). https://www.ligo.caltech.edu/

[61] Maechling, P. et al. SCEC cybershake workflows—automating probabilistic seismic hazard analysis calculations. In *Workflows for e-Science: Scientific Workflows for Grids* (eds. Taylor, I. J., Deelman, E., Gannon, D. B. & Shields, M.) 143–163 (Springer, 2007). 10.1007/978-1-84628-757-2_10

[62] Livny, J., Teonadi, H., Livny, M. & Waldor, M. K. High-Throughput, Kingdom-Wide prediction and annotation of bacterial Non-Coding RNAs. *PLoS ONE*. **3** (9), e3197. 10.1371/journal.pone.0003197 (2008). 18787707 10.1371/journal.pone.0003197 PMC2527527

[63] Sriperambuduri, V. K. & Medishetty, N. Effective workflow scheduling in cloud platform using data aware based adaptive gravitational search algorithm. *Int. J. Innov. Eng. Sci. (IJIES)*. **16** (4), 254–263. 10.22266/ijies2023.0831.21 (2023).

[64] Muller, K. E. & Fetterman, B. A. *Regression and ANOVA: An Integrated Approach Using SAS Software* (John Wiley & Sons, Inc., 2003).

